# First bioanthropological evidence for Yamnaya horsemanship

**DOI:** 10.1126/sciadv.ade2451

**Published:** 2023-03-03

**Authors:** Martin Trautmann, Alin Frînculeasa, Bianca Preda-Bălănică, Marta Petruneac, Marin Focşǎneanu, Stefan Alexandrov, Nadezhda Atanassova, Piotr Włodarczak, Michał Podsiadło, János Dani, Zsolt Bereczki, Tamás Hajdu, Radu Băjenaru, Adrian Ioniță, Andrei Măgureanu, Despina Măgureanu, Anca-Diana Popescu, Dorin Sârbu, Gabriel Vasile, David Anthony, Volker Heyd

**Affiliations:** ^1^ Martin Trautmann, Bianca Preda-Bălănică, Volker Heyd Department of Cultures/Archaeology, University of Helsinki, Helsinki, Finland.; ^2^ Alin Frînculeasa Prahova County Museum of History and Archaeology, Ploieşti, Romania.; ^3^ Marta Petruneac, Marin Focşǎneanu ‘Horia Hulubei’ National Institute for R&D in Physics and Nuclear Engineering (IFIN-HH), Măgurele, Romania.; ^4^ Stefan Alexandrov National Archaeological Institute with Museum (NAIM-BAS), Bulgarian Academy of Sciences, Sofia, Bulgaria.; ^5^ Nadezhda Atanassova Institute of Experimental Morphology, Pathology and Anthropology with Museum (IEMPAM), Bulgarian Academy of Sciences, Sofia, Bulgaria.; ^6^ Piotr Włodarczak Institute of Archaeology and Ethnology, Polish Academy of Sciences, Kraków, Poland.; ^7^ Michał Podsiadło Dolmen S.C., Kraków, Poland.; ^8^ János Dani Déri Museum, Debrecen, Hungary.; ^9^ Zsolt Bereczki Department of Biological Anthropology, Szeged University, Szeged, Hungary.; ^10^ Tamás Hajdu Department of Biological Anthropology, Eötvös Loránd University, Budapest, Hungary.; ^11^ Radu Băjenaru, Adrian Ioniţă, Andrei Măgureanu, Despina Măgureanu, Anca-Diana Popescu, Dorin Sârbu, Gabriel Vasile Vasile Pârvan Institute of Archaeology, Romanian Academy, Bucharest, Romania.; ^12^ Hartwick College, Oneonta, NY 13820, USA.; ^13^ Harvard University, Cambridge, MA 02138, USA.

## Abstract

The origins of horseback riding remain elusive. Scientific studies show that horses were kept for their milk ~3500 to 3000 BCE, widely accepted as indicating domestication. However, this does not confirm them to be ridden. Equipment used by early riders is rarely preserved, and the reliability of equine dental and mandibular pathologies remains contested. However, horsemanship has two interacting components: the horse as mount and the human as rider. Alterations associated with riding in human skeletons therefore possibly provide the best source of information. Here, we report five Yamnaya individuals well-dated to 3021 to 2501 calibrated BCE from kurgans in Romania, Bulgaria, and Hungary, displaying changes in bone morphology and distinct pathologies associated with horseback riding. These are the oldest humans identified as riders so far.

## Teaser

Human skeletal remains indicate the practice of horseback riding as early as the 30th century BCE.

## INTRODUCTION

### Multidisciplinary sources of evidence for earliest horsemanship

Using horses for transport was a decisive step in human cultural development. Trade and cultural exchange as well as conflicts and migrations leapt with the increase in speed and range provided by horsemanship. Archeological, archeozoological, and paleogenetic research into the beginnings of horse domestication and the initial expansion of domesticated horses (*Equus caballus*) has recently seen much progress (*1*, *2*), as has our understanding of the appearance of horse-drawn fast chariots with spoked wheels ~2000 BCE (*3*).

However, information for earliest horseback riding so far is sparse (see section S1 for a detailed review). Possible bit wear in premolar teeth of horses from Botai (Kazakhstan) dating to <3500 BCE were extensively debated during the past three decades (*4*–*6*). Information from the Botai site such as horse demography, horse dung finds, potential paddock fences, or horse milk traces in pot shards (*7*, *8*), as well as horse milk peptides in the calculus of Yamnaya individuals from Krivyanskiy 9 (Russia; ~3000 BCE) (*9*), suggests that domestication became widely established during the second half of the fourth millennium BCE. However, these do not provide direct evidence for riding.

Depictions from the Mesopotamian Ur III period shortly before 2000 BCE may be the earliest figurative evidence for riding, probably on a horse (rider seated forward, falling mane, and bushy tail hair) but possibly on an ass (*Equus asinus*) or ass-onager (*Equus hemionus*) hybrid (*10*). During the Old Babylonian period of the early second millennium BCE, indubitable images and literary mentions in cuneiform texts (*11*) prove that horses were ridden. However, it is apparent that organized cavalry was introduced not before the very end of the second millennium BCE (*4*).

**Fig. 1.**
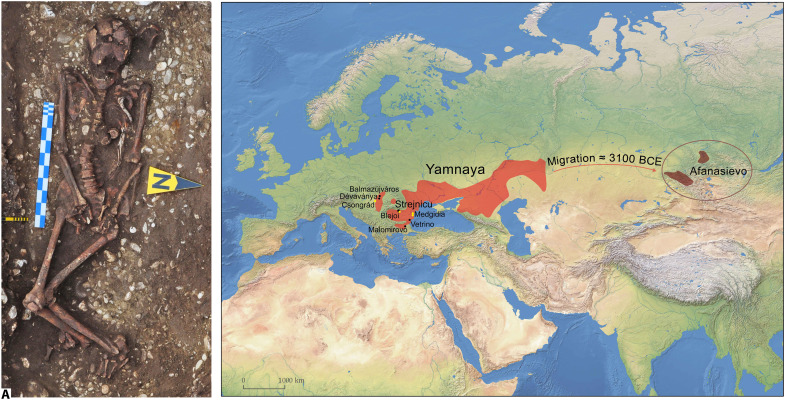
Map of the Yamnaya and Afanasievo overall distribution. Sites with individuals with skeletal markers for horsemanship are marked (black circles, Yamnaya; yellow circles, graves dated to other periods) (the background map is made with Natural Earth; free vector and raster map data at naturalearthdata.com; QGIS software). 1a, Strejnicu mound I grave 3 (I/3) grave in situ (photo credit: A. Frînculeasa, Prahova County Museum of History and Archaeology).

While this provides a rough time frame and geographical setting, our understanding of how horsemanship developed between mid-fourth and early second millennium BCE in the Pontic-Caspian steppe and the Middle East is still vague. This time span also sees the first horse dispersals to the west and south (*1*, *2*, *10*, *12*), the origins of modern horse breeds (*1*), the widespread introduction of cattle-pulled wheeled carts and wagons (*13*), and the Yamnaya (~3200 to 2500 BCE) expansions eastward to the Altai and Mongolia in the form of the Afanasievo culture (*14*) and westward into the southeast of Europe, coming to a hold at the Tisza river in eastern Hungary (*15*). Latest research into this event (Fig. 1) indicates its rapid accomplishment within one or two centuries just before and after 3000 BCE. Considering the vast geographical distances of 4500 km between the Tisza river and the Altai mountains, the absence of roads, and the small overall population sizes, it is difficult to envision how this expansion could have taken place without improved means of transport.

Yamnaya people had horses, as we know from their few settlements, where horse bones ranged widely from 1 to 2% up to 80% of animal bones, as well as from occasional horse bones found in kurgan fillings (fig. S1) [(*16*), pp. 150–157, (*17*)]. What we lack is archeozoological or artifactual evidence for their use, whether as livestock, as beast of burden or mount and draft animal, or as prestige good. However, the quantity and preservation of Yamnaya horse remains are insufficient for biomechanics studies regarding possible use. In addition, specialized riding tack is not essential for riding or can be made from perishable materials only, so its absence in findings is no proof against riding.

## RESULTS

### Osteological evidence for earliest horsemanship: Strejnicu mound I grave 3 and other Yamnaya individuals

Horsemanship has two interacting components: the horse as mount and the human as rider (*18*). Horseback riding is a demanding physical activity, and adaptive changes of the musculoskeletal apparatus as response to frequent specific biomechanical stressors are well documented (*19*–*21*). Human skeletons from archeological sources are available in higher numbers than horse skeletons and often are better preserved because of proper burials. They therefore provide a more approachable source of information about horse riding (see section S2 and fig. S2 for details). There is continuing research in this direction [(*22*), pp. 28–38], but diagnosing an individual as a rider by skeletal traits alone still encounters limitations. The influence of genetics, age, sex, height, weight, limb proportions, diet, or pathological conditions on the relevant skeletal traits is not fully understood; there are no experiments with regard to the thresholds of strain necessary to provoke adaptive responses. Some activities other than riding, such as barrel making or basket weaving, could result in similar biomechanical stress and, thus, similar reactions of bone tissue. Not all possible indicators are equally reliable, and there is no established evaluation system, so interpretations can vary [(*22*), p. 40]. No doubt, a comprehensive basic study on reliable control groups (e.g., Early Neolithic Linearbandkeramik (LBK) versus Early Medieval Eurasian nomads) with proven rider and nonrider background is lacking. However, a number of studies of historical human skeletons with known equestrian activity demonstrate the informative value of a certain set of osteological traits specifically associated with horse riding, on which we base our observations (*23*–*25*).

**Fig. 2.**
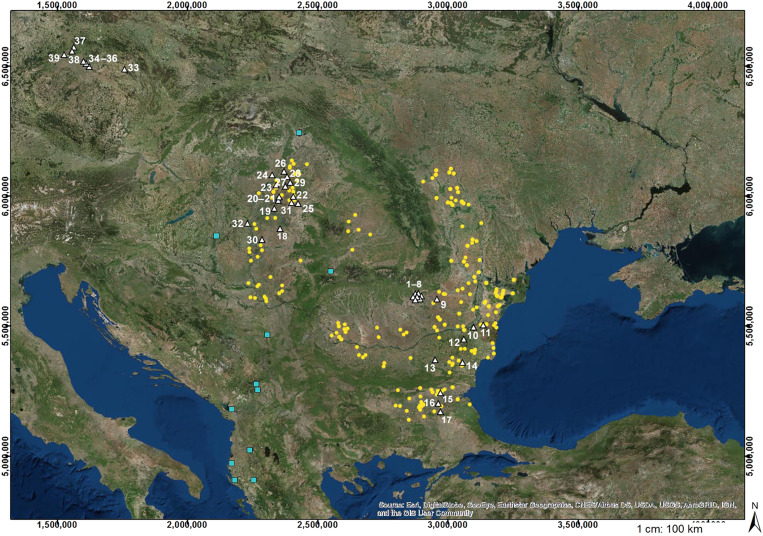
Distribution of the 217 individuals from 39 sites that were bioanthropologically evaluated for this study. The background map displays all excavated Yamnaya kurgans—yellow dot—in Romania (RO), Bulgaria (BG), Hungary (HU), and Serbia (Fig. 9 of *15*, amended); light blue squares denote potential Yamnaya kurgans outside the main distribution. Sites, white triangles: RO 1, Ariceştii-Rahtivani; 2, Blejoi; 3, Coada Izvorului; 4, Nedelea; 5, Păuleşti; 6, Ploieşti; 7, Strejnicu; 8, Târgşoru Vechi; 9, Boldeşti-Grădiştea; 10, Aliman; 11, Medgidia. BG 12, Kamentsi; 13, Chudomir; 14, Vetrino; 15, Mogila; 16, Boyanovo; 17, Malomirovo. HU 18, Kétegyháza; 19, Dévaványa; 20, Sárrétudvari; 21, Püspökladány Kinczesdomb; 22, Debrecen; 23, Balmazújváros; 24, Mezőcsát; 25, Bojt; 26, Tiszavasvári; 27, Nagyhegyes-Elep; 28, Hajdunánás-Tedej; 29, Hajduböszörmény; 30, Földeák; 31, Berettyóújfalu; 32, Csongrád. Czechia (early Corded Ware) 33, Plotiště; 34, Neratovice; 35, Obříství; 36, Vliněves; 37, Trmice; 38, Stadice; 39, Konobrže.

Here, we report five Yamnaya individuals from the sites of Strejnicu in Romania, Malomirovo and Vetrino in Bulgaria, and Dévaványa and Balmazújváros in Hungary. We also briefly discuss two 1750 to 1540 and 1611 to 1446 calibrated BCE Middle Bronze Age individuals from two Medgidia mounds in Romania and the cases of a 3331 to 2927 calBCE “pre-Yamnaya” individual of Blejoi in Romania and a 4442 to 4243 calBCE Copper Age individual of Csongrád-Kettőshalom in Hungary (individual nos. 064, 116, 118, 213, 215, 153, 161, 032, and 209 in Tables 1 and 2). These individuals display ≥4 of 6 (diagnostic threshold of >50%) skeletal traits indicative of the so-called “horsemanship syndrome” (*26*) with a high level of diagnostic certainty. We also list 15 more individuals, of which 9 are Yamnaya, with three positive diagnostic trait categories and, thus, lower probability (Table 1). These were among 217 mostly “steppe” individuals from 39 sites dated between fifth and second millennium BCE studied 2019 to 2022, of which ~150 are archeologically assigned to the Early Bronze Age Yamnaya culture (see Materials and Methods and Fig. 2 for details; see also section S3). Note that recognizing evidence of horseback riding was not the intention of the undertaken study on the mentioned skeletal materials; finding the traits was incidental and rather unexpected.

**Table 1.** List of individuals with possible “horsemanship syndrome” displaying at least three of the six diagnostic traits. Overview of trait appearances (+, present; −, absent; ?, not preserved; numbers in brackets indicate the relative weight of diagnostic specificity).

Ind. no.	032	034	064	081	082	092	103	116	118	130	135	148
Femoral/pelvic entheses (3)	**+**	**+**	**+**	**+**	**+**	−	**+**	**+**	**+**	**+**	**+**	**+**
Ovalization of acetabulum (3)	**?**	**?**	**+**	**?**	**?**	**?**	**?**	**?**	**+**	**?**	**?**	−
Femoroacetabular lesion (2)	**+**	**+**	**+**	**?**	**+**	**+**	**+**	**?**	**+**	**+**	**+**	**+**
Platymeric femur (2)	**+**	**+**	**+**	**+**	**+**	**?**	**?**	**+**	**+**	**+**	−	−
Specific vertebral degeneration (1)	**+**	−	**+**	**+**	−	**+**	−	**+**	**+**	**?**	**+**	**+**
Specific trauma (1)	−	−	**+**	−	−	**+**	**+**	**+**	−	−	−	−
Number of traits	4	3	6	3	3	3	3	4	5	3	3	3
**Ind. no.**	**153**	**161**	**164**	**166**	**170**	**174**	**177**	**186**	**198**	**209**	**213**	**215**
Femoral/pelvic entheses (3)	**+**	**+**	**+**	**+**	**+**	**+**	**+**	**+**	**?**	**+**	**+**	**+**
Ovalization of acetabulum (3)	**+**	**+**	**?**	**?**	**?**	**?**	**?**	**?**	−	**+**	**+**	**+**
Femoroacetabular lesion (2)	**+**	−	**+**	**?**	**+**	**+**	**+**	**+**	**+**	**+**	**+**	−
Platymeric femur (2)	−	**+**	−	**+**	**+**	**+**	**+**	**+**	**?**	−	**+**	**+**
Specific vertebral degeneration (1)	**+**	**+**	**+**	**?**	**?**	**?**	**?**	**?**	**+**	**+**	**+**	**+**
Specific trauma (1)	**+**	−	−	**+**	**?**	−	−	**?**	**+**	**+**	−	−
Number of traits	5	4	3	3	3	3	3	3	3	5	5	4

**Table 2.** List of individuals with possible horsemanship syndrome displaying at least three of the six diagnostic traits. Individual data and relative diagnostic weight in the applied scoring system. Highlighted are the individuals with ≥4 of 6 traits and ≥7 of 12 points. RO, Romania; BG, Bulgaria; HU, Hungary; CZ, Czechia; m, male; f, female; B.P., Before Present.

Ind. no.	Country	Tag	Complete	Sex	Age	Date	Score
**032**	**RO**	**Blejoi (Prahova District, Romania) 2016, mound III, grave 3**	**95%**	**m**	**25–35 years**	**Pre-Yamnaya (DeA-8814) 4437 ± 34 before the present (B.P.), 3331–2927 calBCE**	**7**
034	RO	Blejoi (Prahova District, Romania), mound III, grave 5	100%	m	25–35 years	Pre-Yamnaya (DeA-8815) 4452 ± 33 B.P., 3338–2939 calBCE	5
**064**	**RO**	**Strejnicu (Prahova District, Romania) 2011, mound I, grave 3**	**95%**	**m**	**30–40 years**	**Yamnaya (Hd-30719) 4106 ± 38 B.P., 2869–2501 calBCE (BRAMS-3586) 4190 ± 28 B.P., 2891–2669 calBCE**	**12**
081	BG	Boyanovo (Yambol District, Bulgaria), “Bajlar Kajrak,” mound I, grave 13	85%	m	22–26 years	Yamnaya	5
082	BG	Boyanovo (Yambol District, Bulgaria), “Bajlar Kajrak,” mound III, grave 2	90%	m	35–45 years	Post-Yamnaya	5
092	BG	Boyanovo (Yambol District, Bulgaria), “Bajlar Kajrak,” mound I, grave 17	90%	m	25–30 years	Pre-Yamnaya (early Yamnaya?)	3
103	CZ	Vliněves (Mělník District, Czech Republic), grave 4214A; inv. no.: P7A 41603	95%	m	25–35 years	Corded Ware (CRL-9194) 4133 ± 87 B.P., 2896–2488 calBCE (MAMS-44711) 4174 ± 25 B.P., 2881–2669 calBCE	5
**116**	**BG**	**Malomirovo (Yambol District, Bulgaria) 2021, grave 17**	**90%**	**m**	**65–75 years**	**Yamnaya (Poz-141946) 4315 ± 35 B.P., 3018–2884 calBCE**	**7**
**118**	**BG**	**Vetrino (Varna District, Bulgaria) 2020, Necropole 1, mound XXXIV, grave 3**	**95%**	**m**	**25–35 years**	**Yamnaya (SUERC-95535) 4138 ± 22 B.P., 2873–2623 calBCE**	**10**
130	BG	Vetrino (Varna District, Bulgaria), Necropole 3, mound I, grave 9	80%	(f)	35–45 years	Yamnaya (SUERC-97452) 4172 ± 27 B.P., 2883–2635 calBCE	6
135	BG	Chudomir (Razgrad District, Bulgaria), mound I, grave 9	85%	m	35–50 years	Yamnaya	4
148	RO	Medgidia (Constanţa District, Romania), mound V/VI, grave 1a	100%	m	35–40 years	Post-Yamnaya	3
**153**	**RO**	**Medgidia (Constanţa District, Romania) 2010, mound VI, grave 6**	**100%**	**m**	**40–50 years**	**Middle Bronze Age (DeA-9728) 3254 ± 28 B.P., 1611–1446 calBCE**	**9**
**161**	**RO**	**Medgidia (Constanţa District, Romania) 2010, mound V, grave 4**	**85%**	**m**	**45–60 years**	**Middle Bronze Age (DeA-9667) 3361 ± 32 B.P., 1750–1540 calBCE**	**7**
164	RO	Medgidia (Constanţa District, Romania), mound V, grave 7	95%	(m)	15–18 years	Yamnaya	4
166	RO	Medgidia (Constanţa District, Romania), mound III, grave 1–2, individual 1	70%	m	35–45 years	Pre-Yamnaya	6
170	RO	Medgidia (Constanţa District, Romania), mound III, grave 11	65%	(m)	25–35 years	Yamnaya (BRAMS-3579) 4129 ± 28 B.P., 2870–2581 calBCE	7
174	RO	Medgidia (Constanţa District, Romania), mound III, grave 16	75%	(f)	17–20 years	Yamnaya (BRAMS-3582) 4106 ± 28 B.P., 2865–2505 calBCE	6
177	RO	Aliman (Constanţa District, Romania), grave 1/s	75%	m	40–50 years	Yamnaya (BRAMS-3575) 4096 ± 28 B.P., 2859–2500 calBCE	6
186	HU	Debrecen (Hajdú-Bihar District, Hungary), Basahalom 1906/1326/32	30%	(m)	20–40 years	Yamnaya (DeA-34604) 4322 ± 32 B.P., 3018–2888 calBCE	7
198	HU	Kunhegyes (Jász-Nagykun-Szolnok District, Hungary), Nagyállás-halom grave 18	75%	m	35–50 years	Yamnaya (Poz-39456) 4195 ± 35 B.P., 2895–2636 calBCE	3
**209**	**HU**	**Csongrád (Csongrád-Csanád District, Hungary) 1963, Bárdos-farmstead, -Kettőshalom, grave 1**	**100%**	**m**	**25–35 years**	**Copper Age (Poz-41865) 5470 ± 40 B.P., 4442–4243 calBCE**	**9**
**213**	**HU**	**Dévaványa (Békés District, Hungary) 1969, Barcé-halom, grave 1**	**90%**	**m**	**40–50 years**	**Yamnaya (DeA-8221) 4279 ± 22 B.P., 2916–2881 calBCE**	**10**
**215**	**HU**	**Balmazújváros (Hajdú-Bihar District, Hungary) 1964, Árkusmajor, –Kettőshalom, grave 1**	**40%**	**m**	**35–45 years**	**Yamnaya (Poz-39461) 4320 ± 35 B.P., 3021–2886 calBCE**	**7**

**Fig. 3.**
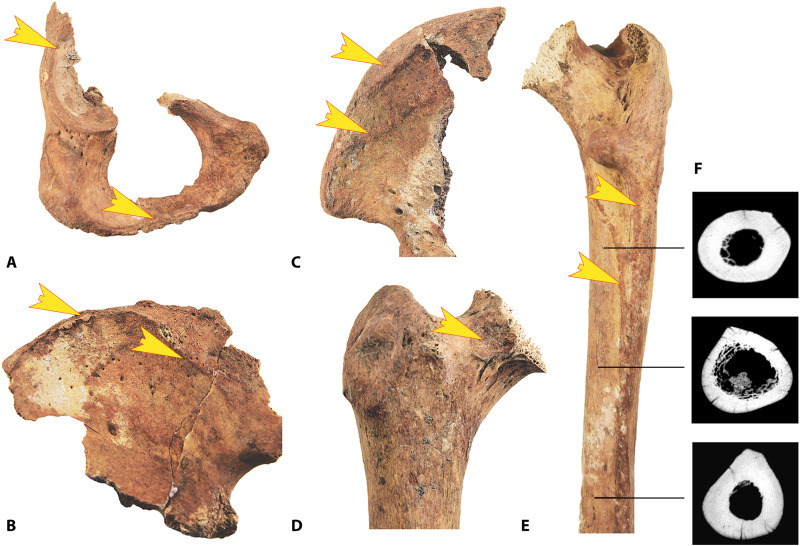
The Strejnicu I/3 individual: Adaptive changes to bone morphology. (**A**) The sclerotic plaque caused by femoroacetabular contact. (**B**) The elevated entheses of the M. adductor magnus and the thickened lateral to superior acetabular rim. (**C**) The entheses of M. iliacus. (**D**) The entheses of M. glutaeus minimus and medius. (**E**) The entheses of M. pectineus, M. adductor magnus, M. adductor brevis, and M. vastus lateralis (photo credit: M. Trautmann, University of Helsinki). (**F**) Femoral shaft cross sections in the computed tomography (CT) (see section S5).

**Fig. 4.**
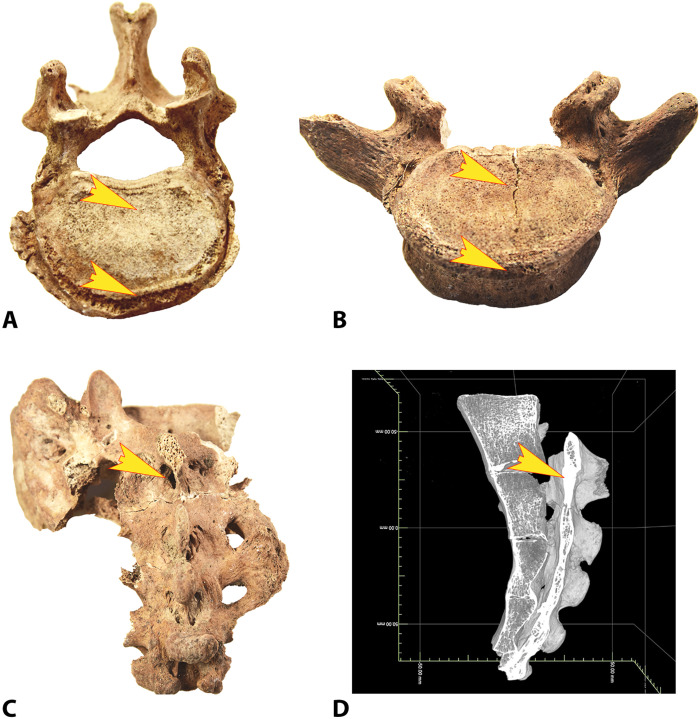
The Strejnicu I/3 individual: Degenerative and traumatic traits. (**A** and **B**) The thickened and sclerotic frontal margin of two vertebrae and the concave deformation of the end plates. (**C**) The misaligned processus spinosus of the first sacral vertebra (photo credit: M. Trautmann, University of Helsinki). (**D**) A CT image of the same feature (lateral view), displaying the replacement of spongious tissue by compact bone (see section S5)

The starting point of our study is the well-preserved skeleton (Fig. 1A) from Strejnicu (Prahova District, Romania), mound I, grave 3 (hence, I/3), dated to ~2879 to 2633 calBCE (see section S4 and figs. S3 to S5) and displaying a typical Yamnaya culture burial (*15*, *27*). The individual, morphologically male, died at an age of 30 to 40 years. With ~165 cm of height, he was rather short compared to other males of the same population (regional mean of ~172 cm) but shared their robust phenotype. Osteological examination showed one of the best records of distinct adaptive, degenerative, and traumatic traits of any of the examined skeletons, thus providing valuable insights about physical activity of the deceased. Although there is no consensus on an optimal set of diagnostic traits [(*22*), p. 40], we recorded the following, which are used widely as indicators of more than occasional horseback riding activity:

1) Entheseal stress reactions on pelvis and femur (Fig. 3, A to C and E): The individual from Strejnicu I/3 exhibits pronounced entheseal marks (*25*, *28*, *29*), especially on the attachment sites of adductor muscles on pelvis and femur, namely, Musculus glutaeus medius and minimus, M. adductor brevis, M. adductor longus, M. adductor magnus, M. pectineus and M. iliacus. They are not completely uniform, but they generally show raised margins, dense and rugose surfaces, and only slight enthesophytes but no cortical defects or porosities. When sitting astride on a moving mount, especially without saddle and stirrups, a rider must hold on to the mount’s back and balance each step by continuous and sometimes forceful contractions of his lower body and thigh muscles. In everyday locomotive activity, these muscles usually experience a continuous but rarely intense workload.

2) Acetabular ovalization (Fig. 3A): The pelvis of Strejnicu I/3 is only partially preserved, so measurements of the acetabulum were not available. Still, the anterosuperior margo acetabuli appears extended, thickened, and very dense, possibly a response to pressure or impact stress inflicted by frequent close contact of this structure with the collum femoris in a sitting position with updrawn legs [(*22*), pp. 85–87, (*30*, *31*)]. In theory, anterosuperior ovalization of the acetabulum could also develop from normal standing and walking activity in individuals with a very high body weight.

3) Femoroacetabular alterations (Fig. 3D): Both femurs of the Strejnicu I/3 individual show distinct impression dents with a dense and raised bony margin on the anterosuperior part of the collum femoris (*32*, *33*). This so-called antero-iliac plaque probably shares its etiology with Poirier’s facet but may be a less-reliable symptom of horse riding [(*22*), pp. 67 and 143). It indicates a frequent and long-lasting sitting position with spread and drawn-up legs. Pincer-like protrusions of the acetabular rim or an asymmetric shape of the collum femoris as connected with the two types of femoroacetabular impingement condition were not observed. The surface is elevated and not cribrous, which rules out Allen’s fossa, and not clearly connected to the articular surface as typical for Poirier’s facet.

4) Bone-shaft cross-sectional shape (Fig. 3F; see also section S5): Both femoral diaphyses display anteroposterior flattening (platymeric index 81.4) and a thicker medial and lateral cortical mass in the subtrochanteric diaphysis (*34*–*36*). This can be understood as an adaptation to mediolateral bending and traction stress on the proximal femur shaft, as in horse riding. Shape adaption due to mechanic demands of this kind mainly develops during adolescence. This makes it probable that the individual from Strejnicu I/3 rode regularly from a young age (*37*). The upper femur-shaft cross-sectional shape is potentially also influenced by relative pelvis breadth and femur angle, because female individuals usually show a tendency toward a higher platymeric index than males.

5) Stress-induced vertebral degeneration (Fig. 4, A and B): The Strejnicu I/3 individual shows signs of a medium-level spondylosis of the lower thoracal and lumbar region, with sclerosis of the anterior rim and small osteophytes. Conditions such as Diffuse Idiopathic Skeletal Hyperostosis (DISH) that may influence the assessment were not observed. The vertebrae are affected symmetrically. Their end plates are slightly concave as a symptom of vertical pressure stress on the intervertebral discs and show small herniation imprints (Schmorl’s nodes). Degenerative changes of the spine are common in premodern individuals, but the general degree of spondylopathies appears comparatively low in the Yamnaya population samples so far examined. In addition, asymmetrical alterations that indicate load with a side preference caused by tool use are rarely observed. The changes found here are more in concert with the repetitive vertical impact stress in an upright position with pronounced lordosis, as typically suffered from riding [(*22*), pp.115 and 159, (*38*, *39*)].

6) Trauma by accident (Fig. 4, C and D): The os sacrum of the Strejnicu individual shows a well-healed but slightly misaligned processus spinosus of the uppermost sacral vertebra. No structural weakening by inflammative, infectious, or neoplastic cause is visible, so a traumatic etiology is probable. A fatigue fracture can be ruled out, because the bone part is positioned too high to be affected by any type of sitting position; a forceful fall on the backside is the most likely trauma scenario. Falls from horseback are the most common cause of injury in conjunction with equestrianism, often resulting in fractured bones of the limbs or the trunk (*40*, *41*). Other common injuries from handling horses are from bites (usually the hands are affected), from kicks (most often resulting in pelvic and thoracal injuries), or from stepping on the foot.

## DISCUSSION

### A scoring system for assessing horsemanship syndrome and earliest horse riding

Each single trait as described above may not be a specific “occupational marker” for horse riding. The simultaneous occurrence of all six traits in the case of the Strejnicu Yamnaya man, however, gives the interpretation of habitual horsemanship a good degree of plausibility. Apart from our threshold of more than half of relevant diagnostic traits (≥4 of 6 traits) related to biomechanical stress from frequent horseback riding, we applied an additional scoring point system to better assess the degree of symptomatic probability between individuals. This is due to the consideration that a syndrome does not necessarily need to display all correlated diagnostic symptoms, but diagnostic reliability increases with the number of positive symptoms, and some symptoms have a higher and more specific diagnostic value than others. This scoring system (Table 2; see also section S3; the table also provides a summary of the other burials) takes the occurrence of certain traits, their severity of expression, preservation, and relative importance [(*22*), p. 159] into account. By doing so, we set a second threshold of ≥7 points out of the possible maximum value of 12 (again, more than half).

Besides Strejnicu, particularly the near-complete skeletons of a man of Vetrino (N1, XXXIV/3; fig. S10), 25 to 35 years old at death and a near-coeval dated to 2873 to 2623 calBCE, scores nearly as high, followed by an earlier (2916 to 2881 calBCE) 40- to 50-year-old man of Dévaványa (Barcé-halom, grave 1; fig. S13). Both display pelvic and femoral entheses, acetabular ovalization, femoroacetabular lesions, platymeric femora, and specific vertebral degeneration but lack fall-related traumata. Two more Yamnaya individuals, still displaying four traits, also meet our scoring threshold of ≥7 of 12 points. Of these, the Malomirovo grave 17 (fig. S9) individual, male and 65 to 75 years old, strongly displays the distinctive characteristics and would possibly score higher, if more relevant skeletal regions would be preserved. The Malomirovo and the Balmazújváros (-Kettőshalom grave 1; fig. S11) individuals represent, with radiocarbon dates of 3018 to 2884 and 3021 to 2886 calBCE, respectively, an early Yamnaya horizon.

We also list four more graves meeting the threshold, which are however non-Yamnaya. While the two mature individuals of Medgidia V/4 and VI/6 (figs. S7 and S8) are securely dated to the mid-second millennium BCE, the 25- to 35-year-old man buried in the late fourth millennium BCE in Blejoi III/3 (fig. S6) is culturally displaying a mixture of local elements with those of either pre-Yamnaya or (chronologically possible) incoming first Yamnaya pastoralists (*15*, *27*). Special attention is deserved by the case of the individual of Csongrád-Kettőshalom in Hungary (fig. S12). Displaying five traits, this 25 to 35 years old scores as high as our five Yamnaya individuals and thus meets our requirements to qualify as a rider with a sufficiently high probability. However, his Copper Age date in the second half of the fifth millennium BCE and his geographical isolation call for caution because we lack comparably assessed skeletons of this period and his special cultural context [(*42*), pp. 249–257; see also section S1 for his Copper Age/Eneolithic context).

A further nine Yamnaya individuals (Table 2), displaying three traits, however, do not meet the requirements set by our scoring system and, thus, fall short for us to plausibly call them riders. The same applies in our set for three more pre-Yamnaya individuals of the late fourth millennium BCE and a Corded Ware man from the Czech Republic (Vliněves, grave 4214A). Besides the men of Medgidia V/4 and VI/6, for which criteria are met, two extra steppe kurgan burials of the mid-second millennium BCE from Bulgaria and Romania also fall short. While we imagine that horsemanship was possibly widespread in the steppes by this time (*43*), our sample demonstrates that the practice may not always show up reliably in all traits, which is to be expected because of the aforementioned varying influences. This prevents us from securely quantifying horse riding in steppe societies of the third and second millennium BCE. However, having five plausible Yamnaya cases from three different countries in southeast Europe, spanning the near entire duration of Yamnaya culture in these regions, may well speak for a wider practice.

**Fig. 5.**
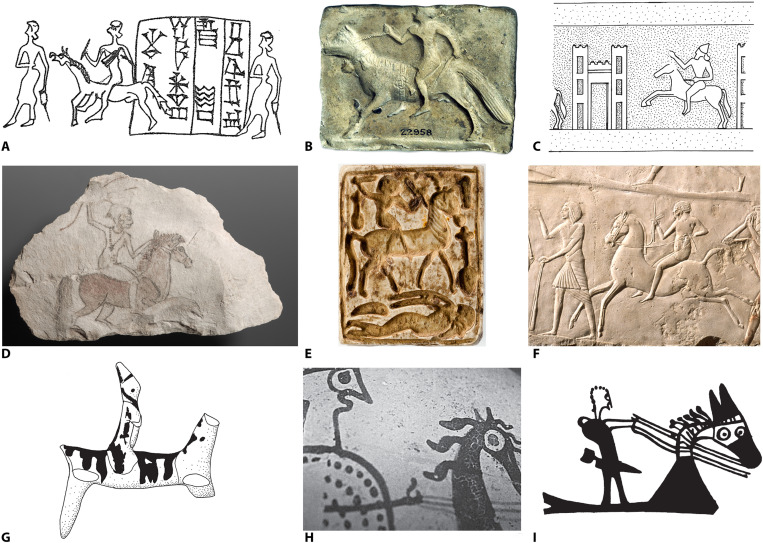
Pictorial evidence of horsemanship in the Bronze Age (c. 2100 to 1200 BCE). (**A** to **C**) Mesopotamia. (**D** to **F**) Egypt. (**G** to **I**) Aegean-Cyprus. (A) Drawing of a seal impression depicting a horse rider, Ur III period (*81*). (B) Baked clay plaque mold depicting a rider, Old Babylonian period [The Trustees of the British Museum; shared under a Creative Commons Attribution–NonCommercial-ShareAlike 4.0 International (CC BY-NC-SA 4.0) license; except where otherwise noted, content within this article is licensed under a CC BY 4.0 license] (*82*). (C) Drawing of a seal imprint of Ili-pada, Middle Assyrian Empire (courtesy of F. Wiggermann, Leiden) (*83*). (D) Astarte on horseback: an Egyptian graffito, Nineteenth Dynasty (photo credit: S. Steiß, Berlin) (*84*). (E) Egyptian plaque of glazed steatite showing a horse rider trampling a fallen enemy, Nineteen Dynasty (The Metropolitan Museum of Art) (*85*). (F) Limestone relief with a messenger on horseback from the Horemheb tomb, Saqqara, Late Eighteenth Dynasty (Museo Civico Archeologico di Bologna) (*86*). (G) Clay figurine of the so-called “cavalryman” from Mycenae, early LH IIIB (courtesy of J. Kelder, Leiden) (*87*). (H) Horseman on an LHIIIB krater in the Allard Pierson Museum, Amsterdam (courtesy of J. Kelder, Leiden) (*87*). (I) Drawing of a sherd showing a horse rider from Minet el-Beida, tomb VI, LH IIIB2 (courtesy of J. Kelder, Leiden) (*87*).

Biomechanical stress markers on human skeletons provide a viable way to further investigate the history of horseback riding and may even provide clues about riding styles and equipment. Depictions of Bronze Age riders (Fig. 5) usually show a position called “chair seat.” This style is mainly used when riding without padded saddle or stirrups to avoid discomfort to horse and rider. It is physically demanding, with the legs exerting constant pressure to cling to the mount’s back and needs continual balancing, but would not preclude activities such as combat or the handling of herd animals, as numerous historical examples demonstrate. The osteological features described here fit well with this riding style and may have been typical for the earliest period of horsemanship. With the later introduction of shaped and padded supporting saddles and stirrups (*28*), other riding styles such as the so called “split seat,” “dressage seat,” and “hunt seat” evolved (see section S2 and fig. S2).

Together, our findings provide a strong argument that horseback riding was already a common activity for some Yamnaya individuals as early as ~3000 calBCE. This supports other tentative third millennium BCE evidence of an early onset of equines as mounts (*44*). However, because of the lack of specialized gear and a comparably short breeding and training history, early horses were probably hard to handle. As Librado *et al.* (*1*) demonstrate, Yamnaya horses were markedly closer to the equid lineage known as DOM2, including all modern domestic horses, than were wild steppe horses from the sixth millennium BCE. When DOM2 was bred from late Yamnaya horses in the steppes during the second half of the third millennium BCE, genes for reduced anxiety/fear response were selected and retained in all later DOM2 horse breeds. Even DOM2 horses can be highly strung and excitable animals, so a still greater anxiety response in early Yamnaya horses probably made them even more likely to “bolt” from violent or loud actions. The military benefit of equestrianism may therefore have been limited; but nevertheless, rapid transport to and away from the site of raids would have been an advantage, even if combat was carried out on foot. Riding certainly was useful for patrolling wide tracts of land and controlling larger herds of livestock (*45*). It consequently would have contributed substantially to the overall success of pastoral Yamnaya society.

## MATERIALS AND METHODS

A large contingent of skeletons from the modern European countries of Romania, Bulgaria, Hungary, and Czechia were bioanthropologically evaluated between 2019 and 2022 in the context of an ongoing multidisciplinary research project centered around Yamnaya burials and kurgans (Fig. 2). Most skeletons are from the Prahova and Constanţa Districts of Romania and the Yambol region of Bulgaria and were investigated during fieldwork in Ploiești (Romania) in 2019, in Yambol (Bulgaria) and Prague (Czechia) in 2020, in Budapest and Szeged (Hungary) as well as in Yambol and Bucharest (Romania) in 2021, and in Sofia (Bulgaria) and Yambol in 2022. Two hundred seventeen individuals from 39 sites were evaluated for the present study. Most (~150) have been assigned to the Yamnaya culture by radiocarbon dating and archeological context of burial customs (*15*, *16*, *27*, *42*), but some individuals are ambiguous or are—as buried in the same burial mound, called kurgan—dated earlier or later, thus belonging to another culture group.

Established osteological macroscopic examination methods were applied to provide standard data for individual and demographic analysis (*46*–*51*). A selection of cranial and postcranial measurements following Howells and Martin for phenotype comparison and possibly kinship analyses (*52*, *53*) were taken, as well as for biomechanical workload analyses. The grade and type of joint surface degeneration and entheseal characteristics (*28*, *54*–*57*) were specifically recorded. Dental status including tooth loss, caries, calculus, periodontal diseases, enamel hypoplasia, dental wear grade and characteristics were documented to allow for dietary reconstruction (*58*–*62*). Furthermore, all observable paleopathological changes, traumata, and activity markers (*63*–*67*) were noted as health status indicators, as well as inheritable morphological variants (*68*, *69*) for studies on kinship and population heterogeneity.

All data were recorded on an adapted recording sheet based on guidelines of the Global History of Health Project and formerly established documentation routines used by the lead author (*50*, *70*). While burials do not usually provide much direct insights into daily life and activities, the remains of the deceased are a rich indirect source of information. Skeletons are archives of past lives with regard to appearance, kinship, health, diet, and activity (*71*–*75*). Taphonomic changes such as discolorations, postmortem defects, animal bite marks, or deformations were documented, and bone and tooth samples for paleogenetic and isotope studies were acquired. High-resolution photos, x-ray, and/or Computed Tomography (CT) scans were taken for a selection of skeletons if necessary.

The burial practice of depositing the body in a timber-covered underground chamber protected by a mound of earth (kurgan) collected from the surrounding surface helped to shield the inhumations against mechanical damage from animals or agriculture but produced increased chemical stress from organic acids originating from decomposition, decaying wood, and rainwater filtering through the cover of loose humus (*70*). Thus, the condition of bone material in general was mediocre. To assess it individually, the preserved percentage of skeletal remains (completeness) and the overall structural integrity (preservation) described by the terms hard, firm, fragile, and brittle was estimated. Values varied depending on factors such as local soil characteristics, specific burial customs, or depth below surface. The overall mean completeness was 57%, with most skeletons showing a firm to fragile condition. For all skeletal individuals, detailed excavation reports and archeological analyses are available (see sections S3 and S4 for the individuals in focus).

Preliminary analysis shows a nonrepresentative demographic selection: 132 individuals were determined as male or probably male, 65 as female or probably female, and 20 were not determined (Masculinity Index 2031). Sixty-one individuals of 217 (28.1%) died at subadult age. The detailed analysis of biomarkers regarding population characteristics and lifestyle indicators awaits the inclusion of further samples and relevant non-Yamnaya population data. However, some trends are already visible: Yamnaya-related individuals show a generally much more robust morphology of cranium and postcranium and a higher mean body height. Dental wear and workload-related joint degeneration correlated to age appear comparatively lower, while muscle attachment marks are more pronounced. Signs of interpersonal violence are very rare. So far, differences in everyday activity patterns and diet are probable and would support the assumption of a transition from a more agrarian pre-Yamnaya Neolithic population to a pastoralist Yamnaya population (*76*–*80*).

## Supplementary Materials

### This PDF file includes:

Supplementary Text Figs. S1 to S13 References
