# Supplementary material for: First bioanthropological evidence for Yamnaya horsemanship
Source: Sci Adv. 2023 Mar 3;9(9):eade2451. doi: 10.1126/sciadv.ade2451 (PMC10954216; doi:10.1126/sciadv.ade2451)
Supplement: Supplementary file 1 — Supplementary Text Figs. S1 to S13 References [file sciadv.ade2451_sm.pdf]

Science Advances
AAAS

# Supplementary Materials for

## First bioanthropological evidence for Yamnaya horsemanship

Martin Trautmann *et al.*

Corresponding author: Volker Heyd, volker.heyd@helsinki.fi; Martin Trautmann, martin.trautmann@helsinki.fi

*Sci. Adv.* **9**, eade2451 (2023)
DOI: 10.1126/sciadv.ade2451

**This PDF file includes:**

Supplementary Text
Figs. S1 to S13
References

**Supplementary Text**

## SM 1 The beginnings of horseback riding, horse skeletal finds in east-central and southeastern Europe, and Yamnaya nomadism

*David Anthony and Volker Heyd*

The beginning of horseback riding was an epochal event in human prehistory because it was the first means of rapid transport, an entirely new kind of human locomotion that transformed warfare and communications. A very recent study of ancient DNA (aDNA) from horse bones across the ancient world *(1)* identified the steppe region between the Don and Ural Rivers as the place where the DOM2 lineage, the ancestor of all modern domesticated horses, evolved over 3000 years between 5500 BCE (wild) and 2200-2100 BCE (the earliest DOM2 horses). Horses from two sites of the Yamnaya culture, Repin on the Don and and Turganik on the upper Samara River, dated 3300-2700 BCE, and one grave of the Steppe Maikop culture, at Aygurskii in the North Caucasus steppes (all Russia), dated 3500 BCE, yielded the pre-DOM2 horse populations that, combined, parented DOM2. We can conclude that Yamnaya horses were managed—successive generations were chosen for the enhancement of the human-accommodating traits that culminated in DOM2. Previously unknown evidence from dairy peptides preserved in dental calculus shows that two Yamnaya individuals from Kryvianski IX on the lower Don had consumed horse milk, as well as cow and sheep milk, a clear sign that Yamnaya horses were domesticated *(9)*.

Yamnaya people from eastern central and southeastern Europe also had horses (*Fig. S1*). Horse bones/teeth were recovered from the earthen fillings of at least four kurgans (sites of Uljma, 'Itebejčeva humka' and Šajkaš, 'Ciganska humka' in Serbia *(88, 89)* and Bashalom-Nagyhalom and Sárrétudavari-Örhalom, Grave 10 in Hungary *(17, 90, 91)*. Of particular interest in this context is the site of Plachi Dol-1 in Bulgaria. Here, bones of *equus caballus* were found next to those of cattle in the filling of the grave pit of central Grave 1/2. The neighboring secondary Grave 1/1, only 4 meters away, is more famous as it yielded the remains of the wheels from the only wooden Yamnaya cart known from southeastern Europe *(92)*. More numerous are however finds of horse bones in roughly contemporary (c. 3500-2700 BCE) settlement sites of the Boleráz, Baden and Coțofeni cultures of the Carpathian basin *(12, 17, 90, 93–95)* and of the Ezero culture in Bulgaria *(96)*. These c. 20-25 sites, and the deposition of complete horses, or larger part of them, in pits of at least three sites *(97)*, in Balatonőszöd-temetői-dűlő and Budaörs-Kamaraerdei-dűlő in Hungary, and Komjatice in Slovakia, demonstrate horses must have widely been known ~3000 BCE. But while most settlement sites only yielded single or a handful of bones, there is also the site of Kiten-Urdoviza beach, a submerged settlement at the Bulgarian Black Sea coast. It culturally belongs to the so-called Ezerovo culture and is dated to c. 3100 BCE, making it roughly contemporary with early Yamnaya. Here, about 450 horse bones/fragments were recovered in an overall collection of several thousand bones *(98)*. Relatively low quantities of horse bones per site may rather speak for horses being no more than individual prestigious items rather than domesticated herds, but sites like Kiten-Urdoviza beach, or the later Kırklareli-Kanlıgeçit in Turkish Thrace (2600-2300 BCE) *(96)* and then the Bell Beaker sites in the area of Budapest (Csepel Háros, Csepel-Hollandi útca,

Albertfalva, Szigetcsép-Tangazdaság I; 2500-2200 BCE) *(99)* demonstrate, with their larger numbers, that proper horse herd management must have taken place too.

But neither aDNA nor dairy peptides can reveal when steppe horses began to be ridden, and it was riding that opened the new era in rapid transport.

The presence of skeletal morphology changes consistent with riding in a Yamnaya individual at Strejnicu I/3 dated 2879-2633 calBCE appears to be one of the oldest such case. Other indicators of riding are more indirect. Wear on horse teeth was attributed to the use of organic bits at Botai in northern Kazakhstan dated 3500-3100 calBCE *(4, 7, 8)* and in Mongolia dated 1200 BCE. Taylor and Barrón-Ortiz (2021) *(5)* argued that some of the dental wear at Botai was not caused by bits, but this is hardly the final word. In any case, the Botai horses belonged to a separate lineage (DOM1) that left only traces of ancestry in DOM2 *(100)*, so Botai was an independent domestication event that involved a distinct horse clade that ultimately was replaced by DOM2 horses.

Little of the equipment of riders survives, but some artifacts have been interpreted as cheekpieces for bits. Dubious claims for Eneolithic antler cheekpieces dated 4000 BCE *(101)* are not much discussed now. The oldest bridle cheekpieces appear to have been looped copper artifacts found in situ with Maikop-culture oxen dated about 3200 BCE *(102)*. The oldest cheekpieces used with horse bridles were carved antler discs found in pairs with chariot horses in the Sintashta and Potapovka cultures, dated after 2000 BCE, but these cheekpieces were used in driving *(3, 103)*. The Yamnaya rider from Strejnicu presents more direct evidence for riding than any of these.

The initial advantages of riding probably were realized in mundane behaviors. In Mongolia, a single mounted herder could manage two to three times more sheep than a pedestrian, with no increase in labor *(45)*. For steppe societies that kept domesticated cattle and sheep for feasts and ritual sacrifices, as at Eneolithic Khvalynsk *(104)*, mounted herding made larger herds possible, and therefore larger feasts and bigger acts of generosity. But the larger herds made *manageable* by horseback riding must be fed, and since fodder was not used among Eurasian steppe pastoralists *(45)*, a larger herd needed to move more frequently to renew its pasture. Maintaining larger herds required increased *residential* mobility.

Pastoral nomadism in the Eurasian steppe was defined by residential mobility—the ability of human herders to move their principal residence (including fire fuel, water, food, and shelter) with their herds. A radical new level of residential mobility was made possible after wagon transport was introduced to the steppes about 3500 BCE. The oldest dated wooden wheels in the steppes are from a Steppe Maikop grave at Sharakhalsun 2, Russia, in the North Caucasus steppes, dated 3336–3105 calBCE (4500±40 BP/GIN-12401) *(102)*. Wheels and wagon-graves diffused across the Pontic-Caspian steppes between about 3300-3100 BCE, according to current radiocarbon dates *(13, 102)*. Early solid-wheeled wagons could carry 1-2 tonnes of cargo *(105)*, making it possible to use a wagon as a mobile home. Oxen trained to pull weight became a valuable new human commodity *(102, 106)*. When wagon transport was combined with mounted herding by the Yamnaya culture, a new nomadic way of life was enabled.

Eneolithic sites in the Pontic-Caspian steppes were limited to the river valleys, ribbons of forest and riverine resources a few kilometers wide and hundreds of kilometers long, cutting through a grassland environment that contained few or no archaeological sites and was little used. Morgunova *(107)* and Shishlina *(108)* mapped 80 riverine Eneolithic settlements in the Volga-Ural-Caspian steppes. All were abandoned during the Yamnaya period. A handful of stratified riverine Eneolithic settlements in the lower Don (Samsonovka, Liventsovka, Razdorske) and Dnieper valleys (Mikhailovka, Generalka 2, Strilcha Skelya) retained ephemeral occupations in the Yamnaya period. These western Yamnaya settlements imply longer-term or more frequent re-occupations and more continuity between the Eneolithic and Yamnaya settlement patterns in the Dnieper-Don steppes. But even here, 95% of Yamnaya sites were kurgan cemeteries, some of which were the first significant monuments created by humans in the arid plateaus between the river valleys. The near-disappearance of settlement sites for 1000 years (3000-2000 BCE) across the Pontic-Caspian steppes implies a continuously mobile, nomadic mode of residence.

Accompanying this increase in residential mobility were significant changes in diet. Dietary isotopes from Yamnaya bones in the middle Volga steppes *(109)* and in the lower Don steppes *(110)* show a change in human diets from primarily C3, riverine forest resources such as deer and fish during the Eneolithic to a mixture of C3 and C4, grassland-adapted resources in Yamnaya individuals *(110, 111)*. The human isotopic results could be explained *(109)* "…if it is assumed that sheep milk and meat contributed the majority of the protein in Bronze Age human diets…" in the Volga-Ural steppes. Wilkin's study of milk peptides in human dental calculus in the Volga-Ural steppes found no milk peptides in Eneolithic teeth, so no evidence for dairying, although cattle, sheep, and goats were eaten; but abundant milk peptides in Yamnaya teeth, including genus-specific peptides from cow, sheep-goat, and horse milk. Dairy foods were suddenly ubiquitous in the Yamnaya period *(9, 112)*. The introduction of wagons, their incorporation in graves, the abandonment of Eneolithic riverine settlements, and the shift from riverine to dairy foods happened almost simultaneously at the end of the fourth millennium BCE, signaling an economic transformation that accompanied an ideological shift *(113)* to the religious beliefs associated with the spread of kurgan funeral rituals.

The interpretation of the Yamnaya economy as the first form of steppe nomadic pastoralism *(15, 16, 27, 42, 108, 114–116)* has been strengthened by this previously unidentified data. The first Eurasian steppe nomads enjoyed a distinct advantage because it was the first time that humans systematically harvested the vast bioenergy stored in the Eurasian grasslands. The Yamnaya nomadic community used cattle, sheep, goats, and horses to convert the bioenergy of the Eurasian steppes into a simplified human diet focused on meat and milk proteins, much simpler than the mixture of fish, wild game, and domesticated animals seen in Eneolithic riverine diets. This total dietary reliance on the meat and milk of a few species of domesticated animals, combined with the double role of cattle and horses as both food sources and trained transport animals, changed the cultural value of domesticated animals, with far-reaching consequences first explored by Sherratt *(106)*. The attempt by Yamnaya nomads to spread their economy and political system beyond the steppes then contributed to significant changes in the relationship between the possession of trained traction animals, the use and value of land, and the development of persistent social hierarchies in Bronze Age Europe *(117, 118)*. Horseback riding played an integral role in all these changes.

## SM 2 Biomechanics of horse-riding and skeletal activity markers

*Martin Trautmann*

Bones adapt to mechanical stress in their micro- and macrostructures to better withstand specific demands of loading and bending: Trabecular strength, density and alignment; cortical density and cortical thickness; long bone shaft diameter and cross-section shape; density and surface relief of muscle, tendon and ligament attachments all respond in a way to improve the bone´s resistance to functional demands *(119–121)*. Basically, the repetitive movements of certain activities provoke corresponding osseous changes, which at least in theory can be used to deduce an individual´s activities or even occupation. In practice it is not this easy; individual characteristics like age, sex, height, mass, limb proportions, muscular strength, neuro-motoric peculiarities as well as the genetic responsiveness of bones to stress vary a lot and influence the outcome of changes. Extrinsic factors like the intensity and duration of mechanic stress, the use of different tools or postures also influence individual biomechanics profoundly *(122–126)*. This of course makes it difficult to prove horse-riding activity from skeletal changes alone, even in cases with a rather obvious set of symptoms typical for the so-called '*horsemanship syndrome*'. Even in a population with common and regular riding activity, not every individual will respond the same way *(127–132)*. Some will show no, only a few or mostly slight changes, others will show a mix with uncharacteristic features, and some may be textbook examples, but were not actually very active riders.

One important fact to consider is that most stressors to bones caused by the posture, impact and bending stress inflicted by horse-riding are not exclusive. Sitting with spread and/ or angled legs can be part of a habitual sitting, squatting or crouching position during rest or work. Muscle pull to pelvis and legs can be caused by walking and running in rough terrain, and compression stress to vertebrae can be inflicted by frequent lifting and carrying of heavy loads. Fractures can be caused by a multitude of accident or conflict scenarios. This is the main reason why not *one*, but a characteristic *set of several different symptoms* (a syndrome) is necessary to justify a presumptive diagnosis of frequent horse-riding *(22, 133–135)*. Also, we applied certain criteria to each of the diagnostic traits related to equestrianism to possibly exclude or at least diminish the influence of alternative causes:

1. Characteristic entheseal marks of femur and pelvis: We only included individuals with strongly pronounced (at least grade 2 on a scale of 0-3) muscular attachment sites of muscle groups with usually limited everyday biomechanical strain but high demands when riding (mostly adductors of the thigh), especially when combined with a marked femoral antetorsion. We did not include individuals where upper body or lower leg entheses were more pronounced to exclude individuals with a generally high response to mechanical load, and also did not include cases with possible alterations from injuries or periosteal inflammations.
2. Vertical ovalization of the acetabula: If possible, we used measurements to objectively quantify a visible deformation of a usual circular shape. Possible alternate causes like extra-heavy bodyweight or frequent carrying of heavy loads are difficult to exclude from the skeleton alone, but our cases did not show higher degrees of osteoarthrosis in the ankle, knee or hip joints which could be expected in such cases.

3. Femoro-acetabular lesions on the Collum femoris: We did not specifically discern between different expressions of contact stress between the femoral neck/ head and acetabular margin like Poirier´s facet and sclerotic “plaques” but would have excluded cases of Allen´s fossa or individuals with femoro-acetabular impingement of the pincer or cam-type.
4. A platymeric index of the upper diaphyseal femoral cross-section: If possible, we used measurements to objectively quantify a visible medio-lateral extension of a more circular shape. In cases with very pronounced entheseal ridges, we tried to take their impact on measurements into account. Unfortunately, we did not have the resources for X-ray or CT assessment in all cases.
5. Vertebral degeneration indicating repeated vertical impact stress: We only included cases where spondylotic changes on lumbar vertebrae were more pronounced than on thoracical or cervical ones; were deformations and sclerotic cortical reactions were symmetric and focused on the anterior vertebral margin and where concave impressions or Schmorl´s nodes documented high vertical impact stress: We did not include individuals that also showed pronounced spondylarthrosis or alterations from injuries or periosteal inflammations.
6. Traumata probably caused by falls, kicks or bites from horses. Since such trauma events cannot be expected to be very characteristic, we took all fractures into account that are typically caused by falls, although a compelling connection to falls from horseback cannot be established. Blunt and crushing trauma not identifiable by being caused by some implements were also included.

While intrinsic individual differences in response to musculo-skeletal stress can be assumed to average out statistically by large enough sample sizes, the extrinsic factors especially in horseback-riding are hard to grasp, since the horse-rider-environment-equipment system consists of many independently interacting components. There is the rider´s physique, the size and body shape of the horse, its gaits, the ground traversed, gear used – and of course, the riding style *(136–139)*.
Riding is not just sitting on a horse; it is a complicated dynamic process that demands perpetual swift reactions to shifts of balance by mount and rider to avoid falls, discomfort or fatigue. The exact character of the necessary movements varies with the activity. Slow and even trail-riding, straight forward high-speed racing, jumping over obstacles or the bursts of speed and hard stops with sudden changes of direction as required in herding and combat do affect horse and rider in very different ways. As most complex technologies, riding evolved and improved through the millennia. Horses were bred and trained for better behavior, more strength, speed and endurance *(1)*; equipment in the form of specialized horse tack with different saddles and stirrups was introduced, and riders improved their techniques for more secure and comfortable seating and better control. While using biomechanical stress markers on human skeletons promises to be a viable way to further investigate the history of horseback riding, it is necessary to take the current framework conditions into account, like the broad-and barrel-chested body shape of wild and early horses, their stronger anxiety response, and the lack of sophisticated riding equipment.
As mentioned in the main text, Bronze Age depictions of riders (*See Fig. 5*) usually show a riding position called ‘chair seat’ (*Fig. S2/a*). In this position, the rider’s pelvis is tilted slightly backwards, his thighs drawn upwards, while the knees are flexed, exerting constant pressure to cling to the mount’s back. The rider often sits more towards the horse’s croup to avoid uncomfortable pressure and chafing between the horse’s spine and the rider’s ischium. This riding style is generally used when riding minimal horse-gear. It represents the earliest period of horsemanship, and the osteological features found with the Yamnaya sample presented here fit well the chair seat riding style. The so-called ‘split seat’ (*Fig. S2/b*), where the rider sits very upright with a pelvis tilt forward, long hanging legs and a deep center of gravity provides a less

strenuous and more stable seating but is uncomfortable for rider and horse and profits a lot from the padded and shaped saddles that were introduced in Greek and Roman antiquity. Xenophon (died 354 BCE) recommended this seat in his treatise on horsemanship *Περὶ ἱππικῆς, part VII*, and indeed, depictions from Antiquity and the Middle Ages (like on the Bayeux Tapestry) often show this type of seat, in later times with long stirrups. This seat may result in less pronounced femoro-acetabular stress markers and entheses of the legs and trunk, but in a heavier load on the spine and therefore higher vertebral degeneration.

The introduction of stirrups in the 1st millennium AD allowed an easier balance and better support from the rider´s legs, as seen in the modern 'dressage seat' or the 'hunt seat' (*Fig. S2/c, d*). Stirrups give the rider better control and synergistic action with the mount's movement, and provide better nimbleness in the saddle, making military use more effective. While still considerable, the biomechanical load to horse and rider is easier and better distributed, so related stress markers can be expected to be different *(136)*. Depending on the length of the stirrups and the weight put into them as well as the position of the feet, the adductors will experience less load than the muscle groups that stretch the leg (*Musculus quadriceps femoris*), and "squatting facets" of the talocrural joint may develop.

## SM 3 Plausible Yamnaya and non-Yamnaya horse-riders: The skeletal record

*Martin Trautmann*

From the sample of 217 (mostly steppe) individuals from 39 sites in Romania, Bulgaria, Hungary and early Corded Ware individuals from Czechia examined so far, 45 individuals died younger than 16 years of age. Since adaptive stress markers cannot be expected to develop clearly before that age, we did not include these individuals in the following considerations. Indeed, none of them displayed any of the symptoms used as indicators for horse-riding practice. Age-dependency in the development of relevant musculo-skeletal stress markers is assumed *(25, 139)*. And while a decrease in severity at higher age was observed in a different studied group *(140)*, our sample includes several individuals of mature and older age.

Regarding the remaining 172 individuals, 24 (~14%, see (*Table 1*) displayed at least three out of the six diagnostic criteria commonly attributed to horse-riding activity:

1. Entheseal marks of femur and pelvis
2. Vertical ovalization of the acetabula
3. Femoro-acetabular lesions
4. Platymeric index of the upper diaphyseal femoral cross-section
5. Vertebral stress-induced degeneration
6. Traumata caused by falls, kicks or bites from horses.

The studied sample includes many poorly preserved skeletons (average completeness 57%). Also, most of the above-mentioned diagnostic traits can be found in parts of the skeletons (like pelvis, upper femur and vertebrae) that showed an especially bad chance of being preserved in the studied material. As a consequence, we should assume the relative number of 'riders' in the adult population was probably higher than represented by the preserved cases, so more than 20% would be a reasonable figure.
In addition, the individuals with '*horsemanship syndrome*' in our sample appear to be almost exclusively anthropologically male (*Table 2*). While the total contingent shows an unbalanced sex ratio (male to female ~2:1) probably due to a sex-biased preselection in burial customs, the imbalance is striking. While physiological differences of men and women could possibly explain a gender bias in the visibility of some of the relevant traits *(140)*, gender role-specific differences in activity seem very plausible. This would be in contrast to comparable but geographically and chronologically different pastoral populations *(140)*.

Beyond just counting the appearance of diagnostic traits in individuals, we also tried to assess the relative diagnostic certainty in each case by a scoring system that takes the number and diagnostic weight of present traits into account. Based on the observations of *Berthon 2019, p. 157-160 (22)*, we weighted the traits 'femoral/ pelvic entheses' and 'ovalization of acetabulum' with a relative weight of 3, the traits 'femoro-acetabular lesion' and 'platymeric femur-shaft cross-section' with 2 and the traits 'specific vertebral degeneration' and 'specific trauma' with 1, while the clear absence of a diagnostic trait was calculated at -1. If a trait could not be examined due to

preservation reasons, it was considered to contribute a weight of 0. In total, a maximum weight of diagnostic evidence of 12 could be reached.
If we take only those cases into account that scored higher than 50% with regards to *both* the number of positive traits (≥4 out of six) *and* points in the applied scoring system (≥7 out of twelve), we still have nine individuals left that meet the requirements of this two-step filter process (*Table 1*).

So, while Strejnicu I/ 3 (Ind. No. 064 in *Table 1, 2*) was our first and may be the best representative of "Yamnaya horse riders" so far, he is by no means a singular case.

**Individual short descriptions of probable "horse-riders":**

1) **Strejnicu 2011, I/3**: male, 30-40 y, 95% complete, firm bone condition, ochre taint on skull and upper face, sternum and ribs, right arm, legs and feet.
Full dentition, no caries; low molar abrasion (level 2-3) but pronounced wear of upper incisors (5).
Low osteoarthrosis (level 0-1) except for lumbar vertebrae and hand (1-2).
Strong entheseal relief (level 2) on all long bones.
Height (*Olivier*) 165 cm (based on humerus and radius only).
Healed but deformed and dislocated processus spinosus of sacrum S4.
Schmorl's nodes and concave vertebral endplates of lower lumbar vertebrae.
Pronounced and demarcated femoro-acetabular lesion, rough and thick entheseal ridges on femur and pelvis as well as patella; platymeric index 81.4, pronounced femoral antetorsion (collum-condylar angle wider than 20°).

2) **Blejoi 2016, III/3:** male, 25-35 y, 95% complete, firm bone condition, no ochre taint on bones.
No intravital tooth loss, superficial approximal caries 16 and 38; rather uniform medium dental abrasion (level 2-3).
Low osteoarthrosis (level 1) except for thoracal vertebrae and hand (2).
Low to medium entheseal relief (level 1 upper arms and lower legs, 2 on femurs and forearms).
Height (*Olivier*) 166 cm (based on humerus, ulna and femur).
Surface scarring in Sinus maxillaris (chronic Sinusitis maxillaris?); deformed Processus spinosus of sacrum S1 (healed fracture (greenstick fracture?); concave vertebral endplates of lumbar vertebrae.
Pronounced and demarcated femoro-acetabular lesion, rough and thick entheseal ridges on femur.

3) **Medgidia 2010, V/4:** male, 45-60 y, 85% complete, firm bone condition, no ochre taint on bones.
No intravital tooth loss, no caries; rather uniform low to medium dental abrasion (level 3 frontal, 2 sides).
Very low osteoarthrosis (level 0-1).
Strong entheseal relief (level 2) on all long bones except for lower legs (1).
Height (*Olivier*) 180 cm (based on ulna, radius, femur).

Healed fracture of Os nasale. Schmorl´s nodes on several thoracical and lumbar vertebrae and concave vertebral endplates on lumbar vertebrae; no pronounced demarcated femoro-acetabular lesion, rough and thick entheseal ridges on femur. Pronounced femoral antetorsion (collum-condylar angle wider than 20°), platymeric index 73.5, ovalized acetabula (no measurements); squatting facets.

4) **Medgidia 2010, VI/6:** male, 40-50 y, 100% complete, firm bone condition, no ochre taint on bones.
No intravital loss of teeth, no caries; rather uniform low to medium dental abrasion (level 2-3).
Very low osteoarthrosis (level 0-1) except for lumbar vertebrae (2-3).
Very strong entheseal relief (level 3) on all long bones except for lower legs (1).
Height (*Olivier*) 176 cm (based on humerus, ulna, radius, femur, tibia, fibula).
Healed fissure (chondropathia) in left Fovea glenoidalis scapulae; slightly concave vertebral endplates of thoracic and lumbar vertebrae, spondylotic changes to anterior vertebral rim, Schmorl´s nodes.
Demarcated femoro-acetabular lesion, rough and thick entheseal ridges on femur, with more vertical orientation and on pelvis. Pronounced femoral antetorsion (collum-condylar angle wider than 20°), platymeric index 100, ovalized acetabula (52 to 56 mm); squatting facets.

5) **Malomirovo 2021, Grave 17:** male, 65-75 y, 90% complete, firm to fragile bone condition, ochre taint on skull.
Intravital loss of at least five teeth, no caries; rather uniform medium dental abrasion (level 3), seems low for age.
Low osteoarthrosis (level 1) except for vertebrae, shoulder and hand (2-3).
Strong entheseal relief (level 2-3) on all long bones except for lower legs (1).
Height (*Olivier*) 174 cm (based on humerus, ulna, radius, fibula, tibia).
Healed fracture of left 6th (?) rib; healed crushing fracture of medial/distal phalanx of 2nd right toe with resulting ankylosis; healed crushing fracture of medial/distal phalanx of 2nd left finger with resulting ankylosis; concave vertebral endplates of second lumbar vertebra.
Pronounced rough and thick entheseal ridges on femur with strong medial orientation and on pelvis; squatting facets.

6) **Vetrino 2020, Necropole 1, XXXIV/3:** male, 25-35 y, 95% complete, firm bone condition, ochre taint on upper skull, lower legs and feet, possibly on upper body.
Complete dentition, no caries; rather uniform low dental abrasion (level 1-2), seems low for age.
Very low osteoarthrosis (level 0-1) except for hips (2).
Strong entheseal relief (level 2) on all long bones, upper legs even stronger (3).
Height (*Olivier*) 180 cm (based on humerus, femur, tibia).
Chronic stress to vertebral endplates of thoracic and lumbar vertebra (granular surface, non-inflammative discitis), spondylotic changes to anterior vertebral rim, concave vertebral endplates, Schmorl's nodes.

Pronounced and demarcated femoro-acetabular lesion, pronounced rough and thick entheseal ridges on femur and pelvis; platymeric index 68.4, ovalized acetabula (55 to 58 mm); squatting facets.

7) **Balmazújváros 1964, –Kettőshalom, Grave 1:** male, 35-45 y, 40% complete, firm to fragile bone condition, no ochre taint on bones.
No intravital tooth loss, no caries; low molar abrasion (level 2), pronounced wear of frontal teeth (3-4).
Low osteoarthrosis (level 1) except for cervical and lumbar vertebrae and hips (2).
Strong entheseal relief (level 2) on all long bones except humeri (1).
Height (*Olivier*) 178 cm (based on femur).
Chondropathia retropatellaris I (L), Discitis (CV 3-5), Schmorl´s nodes on several lumbar vertebrae and concave vertebral endplates; signs of periostitis on several ribs (interior side; pleuritis?), Os scaphoideum R articular cyst.
No demarcated femoro-acetabular lesion, rough and thick entheseal ridges on femur and on pelvis. Platymeric index 73.0, ovalized acetabulum L with thickened superior rim (no measurements); no squatting facets.

8) **Csongrád 1963, –Kettőshalom, Grave 1:** male, 25-35 y, 100% complete, firm bone condition, ochre taint on lower arms, pelvis, upper legs and knees, feet.
No intravital loss of teeth, no caries; rather uniform low dental abrasion (level 1-2).
Low osteoarthrosis (level 0-1) except for elbows (2).
Very strong entheseal relief (level 3) on all upper long bones, (2) on legs.
Height (*Olivier*) 183 cm (based humerus, ulna, radius, femur, tibia, fibula).
Healed fracture of right ulnar olecranon (Schatzker type III); concave vertebral endplates of lumbar vertebrae with deep hernial impressions L5/S1, spondylotic changes to anterior vertebral rim on thoracic and lumbar vertebrae.
Demarcated femoro-acetabular lesion, rough and thick entheseal ridges on femur and on pelvis. Pronounced femoral antetorsion (collum-condylar angle wider than 20°), platymeric index 100, ovalized acetabula (55 to 59 mm); slight squatting facets.

9) **Dévaványa 1969, –Barcé-halom, Grave 1**: male, 40-50 y, 90% complete, firm bone condition, no ochre taint on bones.
No intravital loss of teeth, no caries; rather uniform medium dental abrasion (level 2-3).
Low osteoarthrosis (level 0-1) except for lumbar vertebrae, elbows and feet (2).
Strong entheseal relief (level 2) on all upper long bones, (3) on legs.
Height (*Olivier*) 174 cm (based on humerus, ulna, radius, femur, tibia, fibula).
Unclear fragment of partly healed bone part with crushing fracture, possibly patella; deep Schmorl´s nodes on several lumbar vertebrae.
Demarcated femoro-acetabular lesion, rough and thick entheseal ridges on femur and on pelvis. Pronounced femoral antetorsion (collum-condylar angle wider than 20°), platymeric index 77.8, probably ovalized acetabula (no measurements); no squatting facets.

## SM 4 Yamnaya archaeology of the Strejnicu I/3 burial and of the other graves displaying '*horsemanship syndrome*'

### 1. Strejnicu (Romania) 2011, Mound I, Grave 3 (I/3) (*Fig. S3-S5*)

*Bianca Preda-Bălănică, Alin Frînculeasa*

During the 3rd millennium BCE a new burial ritual emerged in Caspian-Pontic steppe area, consisting of central graves under kurgans, the supine with raised knees posture of the deceased, ochre staining on grave floors and the body, north-eastern to eastern body orientation, or western in other regions, the predominance of male graves, and arranging the rectangular burial chamber with mats and wood *(42, 108, 141)*. These burials were connected to Yamnaya pastoralists who, according to current research, migrated in two opposite directions. The eastwards migration, now supported by aDNA studies *(142)*, leaped over a considerable distance, reaching the Siberian Altai and the Minusinks Basin of the Middle Yenisej River, with few outlier burials in Mongolia *(14)*. In these regions, starting with the 31st century BCE new burial practices, material culture and economies emerged, assigned to the Afanasievo culture *(143, 144)*. The similarity with Yamnaya burials consists of central burials under mounds made of earth and stones, in some cases surrounded by stone rings, stone or wood covers of the pits, supine with raised knees posture of the deceased; westwards orientation, and ochre staining; grave goods include pottery with geometric ornamentation and rounded or ovoid bases as well as arsenical copper objects, showing links with the Volga-Ural variety of Yamnaya archaeological record *(14, 145)*. Few burials were also found on the way, in Kazakhstan *(14, 42)*.

In the opposite direction, the Yamnaya burials spread quickly across the entire north-west Pontic steppe, and in the plains of south-eastern Europe, reaching their westernmost expansion at the Tisza river in the Hungarian Plain *(27, 42, 146)*. Here, typical burials were found in several hundred kurgans excavated out of thousands such funerary monuments covering the landscape north and south of the Lower Danube, in Thrace and the Pannonian Plain *(15)*. This migration, also supported by recent aDNA studies *(147)*, is now securely dated at the end of the 4th and beginning of the 3rd millennium BCE by tens of high-resolution $^{14}$C dates from kurgans in the modern countries of Romania, Bulgaria, Serbia and Hungary *(27, 148–150)*. The Yamnaya ritual in its westernmost distribution region shows steppe originated practices of central burials under kurgans, the predominance of male graves, the supine with raised knees posture, west-east orientation, ochre staining, arranging of burial chamber with mats and wood, and grave goods usually limited to local vessels, hair rings and necklaces made of animal teeth, the latter especially in children burials *(15, 27, 146, 151, 152)*.

In the context of recent archaeological research conducted in south-eastern Europe, the *Prahova District* located in northern Muntenia, Romania, stands out as one of the most thoroughly investigated micro-regions, providing a complex picture of the interactions and developments taking place before and following the Yamnaya migration. Several hundred burial mounds were mapped in this region (*Fig. S3/a*) *(153)*, still visible in the landscape of this region, where the Romanian Plain meets directly the Carpathian Mountains (*Fig. S3/b-d*). Out of these, 30 were excavated in the past years, containing 70 prehistoric graves with at least 100 individuals *(154)*.

The earliest kurgans were raised during the last third of the 4th millennium BCE, showing a mixture of steppe and local traits, as well as a distinctive burial ritual, with richly furnished collective burials, containing pottery, copper flanged axes, copper torques and spectacle shaped pendants, silver hair rings *(155)*. The arrival of Yamnaya groups at the beginning of the 3rd millennium BCE – also clearly shown in the stratigraphy of the mounds – brought along a change in burial practices. The collective burials along with most of the grave-goods vanished, leaving behind a normative austerity only rarely eluded by the already mentioned presence of hair rings made of precious metals, pots or necklaces made of animal teeth, placed now in most often in individual graves usually containing the remains of adult men, although sub-adult burials should not be ignored. As everywhere in the steppe, ochre became an essential element of the burial ritual, along with the supine with raised knees posture of the deceased and westwards orientation *(27)*.
Our bio-anthropological analysis is focused on the individual uncovered in 2011 in Strejnicu I/3, in the above described region of northern Muntenia. The kurgan, about 1.2 m high and 40 m in diameter at the time of the excavation (*Fig. S4/a, c*), was located in the Ploiești Plain, not far away from the Carpathian Mountains. Three graves were excavated, partially in clear stratigraphical superpositions:

Grave 1 *(156)*, dated to c. 11/13th centuries AD, consisted of the remains of a horse skeleton associated with iron artefacts. The osteological remains were found not far from the centre of the mound, in 40-50 cm of depth, the outline of the grave pit was not visible, and the orientation was on the ENE-WSW direction. Only the skull and parts of the limbs of the horse were found, below the level of carpus and tarsus bones. The horse skull was seated on fragments of the lower front legs, while the lower back legs were placed parallel to each other, about 0.60 m ENE of the skull (*Fig. S4/b*). Grave goods were comprised of an iron arrowhead placed on the left metacarpal, the iron horse bits that were still in the horse's mouth and another badly preserved iron artefact, placed at the base of the skull, directly on the ground.

Grave 2 was a strongly disturbed burial found in the center of the mound, about 0.60 m north of Grave 1. It was identified in between 30-40 cm of depth and it consisted of fragments of the lower limbs of an adult individual (*Fig. S4/b*). It is not clear when this burial was added to the mound, the $^{14}C$ dating failed twice due to lack of collagen in the sample. However, given the considerable distance from Grave 1, the ritual characteristics such as the west-east orientation, the lack of funeral equipment, we are inclined to consider this as a secondary burial dated to the Bronze Age and not connected to Grave 1 *(156)*.

Grave 3, our Yamnaya rider, is the main burial, found in the central area of the mound, in a rectangular pit with rounded corners, 1.90×0.90 m in size, and 0.5 m deep from the ancient surface under the mound. The adult male individual was laid in a typical Yamnaya posture, supine with the upper limbs stretched along the body and the lower limbs initially raised at the knees and later fallen slightly to the right, west (head)-east oriented *(27)* (*Fig. S5/a*). No grave goods were found. Following the excavation, one $^{14}C$ date was made in 2013 in Heidelberg and indicated the interval (Lab ID Hd-30719) 4106±38 BP = 2869-2501 calBCE at 2σ (95.4% probability) *(27)* (*Fig. S5/b*). After the identification of diagnostic markers of horsemanship on the bones of this individual we decided to repeat the analysis and sent a second sample to the Bristol laboratory, which confirmed the results obtained in the first place; respectively (BRAMS-3586) 4190±28 BP = 2891-2669 calBCE at 2σ (95.4% probability) (*Fig. S5/c-e*). All dates were calibrated with OxCal v4.4.2 Bronk

Ramsey and Lee (2013) *(157)*. The attempt to conduct genetic analyses was not successful, as the bones did not yield high amounts of aDNA.

## 2. Blejoi 2016, III/3 (*Fig. S6*)

*Alin Frînculeasa, Bianca Preda-Bălănică*

The archaeological research of Mound 3 in Blejoi (Prahova County) was carried out in 2016 *(158)* to prevent its destruction by the construction of a road. At the beginning of the excavations the mound was in a precarious state of preservation, being affected by intensive agricultural works that led to its flattening. It was 0.6 m high and about 30 m in diameter.

Grave 3 is the primary grave, over which the original mound was built (*Fig. S6/c*). The pit, dug from the ancient surface level into the natural layer of gravel, was oriented in the SW-NE direction and had a rectangular shape with rounded corners. In the upper part it had the following dimensions: length 1.95 m and width 1.13 m, depth 0.60 m, however, it narrowed towards the bottom. The natural gravel excavated and deposited near the pit was visible on its western and northern sides. The deceased, a robust individual, was found in a good state of preservation. It was SW (head)-NE direction, placed in a crouched position on the left side. The upper limbs were bent and brought towards the face and the lower limbs were flexed (*Fig. S6/b, d*). Traces of ochre were found in the area of the neck and upper limbs of the deceased. The grave goods consisted of two silver hair rings and a well-preserved necklace comprised of several ornaments: one copper spectacle-shaped pendant, four copper tubular beads, ten *Dentalium* tubular beads, and seventeen whole kaolin / talc beads. The absolute age, determined on the basis of radiocarbon dating of the human bone, is (DeA-8814) 4437±34 BP, calibrated to 3331-2927 cal BCE (95.4% of probability) (*Fig. S6/a*).

## 3. Medgidia 2010, V/4 (*Fig. S7*)

*Anca-Diana Popescu, Dorin Sârbu, Radu Băjenaru*

The excavation of the Medgidia Mounds V and VI, located next to each other, in the Peștera commune, Constanța District, Romania, on the route of the Cernavodă – Medgidia sector of the A2 highway was carried out in 2010 *(159)*. Mound V was approximately round in shape, with a diameter of about 60 m on the east-west direction and about 52 m on the north-south direction. It had a preserved height of about 2.40 m measured from the south, and 2.80 m measured from the north-east, from approximately 10 m distance from its periphery (*Fig. S7/a-c*). A total number of eight graves were investigated, seven of which displayed the typical Yamnaya burial ritual. Grave 4 was found in the south-eastern sector of the mound, under the stratigraphic baulk separating sections S.II and S.III. The level from which the grave was excavated could not be identified, the feature became visible at a depth of 2.20 m from the upper part of the baulk. The grave pit was rectangular with rounded corners, 1.68×0.94 m in size, with the long side oriented on the west-east direction. On the pit surface, in the gray layer, at approximately 0.20 m from its western,

northern and eastern limits, a reddish-brown stripe appeared. These were the remains of the vegetal material in which the deceased was wrapped and laid inside the pit. The individual was found at a depth of 2.34 m, crouched on the left side, with the arms flexed and brought towards the face and the legs bent (*Fig. S7/d*). It was east (head)-west (1400‰ E) oriented, facing south. Some bones were reddish in color, probably due to the contact with the material in which the body was wrapped. In the area of the pelvis a bone arrow with three edges was found, that finds analogies in the North-Pontic area (*Fig. S7/e*). The absolute age, determined on the basis of radiocarbon dating of the human bone, is (DeA-9667) 3361±32 BP = 1750-1540 calBCE at 2σ (95.4% probability).

4. **Medgidia 2010, VI/6 (*Fig. S8*)**

*Andrei Măgureanu, Gabriel Vasile, Despina Măgureanu, Adrian Ioniță*

This mound *(159)* was about 1.28 m high and 70×81.5 m (EW-NS) in diameter at the time of the excavation (*Fig. S8/b*). Feature 8/Grave 6 was a secondary grave identified as an agglomeration of limestone boulders in the sourthern periphery of the kurgan. At a depth of -1.40 m, the outline of a roughly rectangular pit, 2.20×1.60 m in size (NS-EW), in fact the upper part of a stepped pit was identified. At -1.69 m the pit narrowed, changing its shape and orientation into an oval-shaped, 1.70×1.10 m in size structure. At -2.29 m a reduction was observed, the actual mortuary chamber having dimensions of 1.30×0.80 m. The total depth of the structure, starting from below the stone level to the pit bottom was 1.50 m (*Fig. S8/c*). The adult male individual was laid in crouched position, ending up on the stomach, with arms bent at the elbow and hands under the abdomen. The legs were strongly flexed, the right patella touched the elbow of the right arm at the level of the abdomen (*Fig. S8/a, d*). The skull was turned to the left. The individual was ESE(head)-WNW oriented, facing south. This position suggests the body was tied up. A pot was placed behind the deceased's body, immediately next to the left humerus. The absolute age, determined on the basis of radiocarbon dating of the human bone, is (DeA-9728) 3254±28 BP = 1611-1446 calBCE at 2σ (95.4% probability).

5. **Malomirovo 2021, Grave 17 (*Fig. S9*)**

*Piotr Włodarczak, Michał Podsiadło*

The excavation of the mound on the "Pamukli bair" hill in Malomirovo, Yambol District, Bulgaria, was carried out in 2021 *(160)* as part of a Polish-Bulgarian collaboration. The kurgan was about 3 m high and 40 m in diameter at the time of the excavation. Grave 17 was related to the second construction phase and was dug into the small mound of the earliest phase up to the depth of the bedrock (4.2 m below the top of the mound). The rectangular burial chamber, 2×1.25 m in size, and 1 m deep, was partially enclosed with stones in the upper part. On the northern and western sides of the grave three anthropomorphic stelae were placed (*Fig. S9/a, c*). The chamber was covered with 13-15 longitudinally arranged wooden planks and stones. A rectangular mat intensely colored with ochre covered the pit bottom, especially visible in the western part, red dye being

also used to decorate it with zigzag lines. On the mat, a male individual about 70 years old was laid in a typical Yamnaya posture, supine with the upper limbs stretched along the body and the lower limbs raised at the knees, south-west (head)-north-east oriented. The skull and bones of the feet were intensely colored with ochre (*Fig. S9/b*). Two silver hair-rings were found near the skull. The absolute age, determined on the basis of radiocarbon dating of the human bone, is (Poz-141946) 4315±35 BP = 3018-2884 calBCE at 2σ (95.4% probability).

## 6. Vetrino 2020, Necropole 1, XXXIV/3 (*Fig. S10*)

*Stefan Alexandrov, Nadezhda Atanassova*

The research of Mound XXXIV, part of Necropolis 1 in Vetrino, Razgrad District, Bulgaria was carried out in 2020 *(161)* as part of a large-scale campaign of rescue archaeological excavations along the expansion of the gas-transmission infrastructure of "*Bulgartransgaz EAD*" company in North Bulgaria. The kurgan was about 0.40 m high and 40 m in diameter at the time of the excavation. A ring with a diameter of 26–27 m, constructed with medium-sized stones arranged in a single row, 1.0–2.5 m in width, surrounded it. Grave 3 was a secondary burial dug into the already existing barrow fill and virgin soil, 3.5 m west of the central point. A rectangular surface of approximately 2.5×1.5 m was dug into the barrow fill to a depth of -0.85 m from the central point. From that level, the rectangular grave pit narrowed to 1.35×0.83 m, and was dug to a depth of -1.05 m from the central point. On the pit bottom, an adult male was laid in relaxed hocker position on the right side, oriented in south-east (head)-north west direction, and facing east (*Fig. S10/f-g*). The left arm of the individual was bent at the elbow, the right arm alongside the body, both palms being placed in front of the pelvis. The lower limbs were bent at the knees. Red ochre had been sprinkled over the feet and palms. As grave goods, two silver hair-rings were found near the skull, one of them in the auricula (*Fig. S10/a-b*); a bone bead and a fragment of a copper/bronze wire with rounded cross section were recovered from the chest area (*Fig. S10/c-d*). A potsherd decorated with cord impressions was found in the fill above the grave (*Fig. S10/e*).
The absolute age, determined on the basis of radiocarbon dating of the human bone, is (SUERC-95535) 4138 ± 22 BP = 2873-2623 calBCE at 2σ (95.4% probability).

## 7. Balmazújváros 1964, –Kettőshalom, Grave 1 (*Fig. S11*)

*Volker Heyd*

This kurgan was situated by the Arkus stream in the Hortobágy steppe of Hajdú-Bihar County. Excavations took place in July 1964 by Gy. Gazdapusztai *(162–165)*. A major part of the kurgan was already levelled before the excavation and only an earthen cone of 6.5 m diameter and 3.2 m high was left standing. The primary Grave no. 1 was found near the supposed centre of the mound first as a rectangular, W-E grave pit with rounded corners. At its bottom, in 4.6 m below the top of the leftover cone, laid a W (head) – E orientated skeleton in the typical supine body position with flexed, originally upright legs, having now tumbled to the left (*Fig. S11*). Its arms were laid

out straight, with the hands placed near the pelvis. The face was looking towards E. Traces of organic grave cover were preserved near the legs and pelvis. Additionally, 'the skeleton was lying on a small postament-like clay bench covered with traces of red paint'. Narrow black stripes, 1-2 cm wide, were visible in the W and around the head. No equipment, except of a small piece of ochre near the left shin, was present. However, at the level of the original surface, directly over the grave which was also covered with some kind of reed and grass, a little grey-brownish mug with a handle ('Töpfchen') and, two meters away, an animal bone, supposed to be from cattle, were found but are now lost.

The absolute age of the individual, determined on the basis of radiocarbon dating of a human bone piece, is (Poz-39461) 4320±35 BP = 3021-2886 cal BCE at 2σ (95.4% probability). A maxilla M2 was also used for strontium analysis, yielding $^{87}Sr/^{86}Sr$ values of 0.70953 for enamel and 0.70991 for dentine.

## 8. Csongrád 1963, –Kettőshalom, Grave 1 (*Fig. S12*)

*János Dani, Tamás Hajdu*

In the western periphery of Csongrád – a town situated at the right bank of the river Tisza - , on the top of a natural loess ridge running in a north-south direction, in 1963, during the rescue excavation relating to sand mining of K. Nagy a Prehistoric solitary burial came to light *(166, 167)*.

According to the original excavation documentation, Grave 1 is oriented SE-NW, with the skull slightly supported, facing east. It was excavated at the northernmost point of the sand mound, but no traces of an earthen barrow above the grave could be identified. The outline of the grave pit was not observed, however the shape of its bottom (in a depth of -1.78 m) was indicated by red ochre sprinkle. The deceased was laid in a supine position, with characteristic raised legs at the knees; the flexed arms were laid parallel to the body with the hands resting on the thighs (*Fig. S12/a*). A 13.2 cm long obsidian blade with trapezoid profile turned up between the upper right arm and the rib cage; 5 beads made of coiled copper plate, lots of little cylindrical spondylus(?) and stone beads and 5 bigger cylindrical stone beads were found around the head and shoulders and around the raised legs (*Fig. S12/b*). A discrete clump of red ochre was more pronounced near the left pelvis *(163, 168, 169)*.

The absolute age, determined on the basis of radiocarbon dating of the human bone, is (Poz-41865) 5470 ±40 BP = 4442-4243 calBCE at 2σ (95.4% probability) *(168)*. This dating corresponds well with the age of the local ECA (Tiszapolgár) communities.

## 9. Dévaványa 1969, –Barcé-halom, Grave 1 (*Fig. S13*)

*János Dani, Tamás Hajdu*

The site of Barcé-halom (Site ID: 4030; it is labelled as 3/49 in the MRT 6 *(170, 171)*, its geographical location in WGS' 84: N47° 02' 29,78" ; E20° 55' 25,14") was excavated in 1969 by I. Ecsedy. He estimated that the possible (original) size of the tumulus could be about 6 m high

and 70 m in diameter (*Fig. S13/a*) *(170, 171)*. However, according to the more recent survey made by Á. Bede, the Barcé-halom tumulus is 5.2 m high and 85×120 m in diameter *(172)*. During the sounding excavation I. Ecsedy made valuable stratigraphical observations: the mound has 2 main periods of construction *(163)*. The first (base) mound was up to 2.10 m high, but the excavation did not reveal the original central burial. After the first construction phase, Grave 1 was cut into this basic mound as a secondary burial. Based on the observation of Ecsedy, from the top of this first kurgan a shaft of 3.6 m diameter of this grave was deepened to the original surface. The whole surface of the shaft's bottom (and the surface of the grave-pit, as well) was covered with white calcified mat remains. The rectangular grave-pit with rounded corners, 1.57×1 m in size, was dug from this depth, cutting the ancient humus layer. The body of the deceased was lying on a surface covered with some kind of organic material, in a supine position, west(head)-east oriented. Probably, the legs were originally raised at the knees, but after the decomposition of the body both legs leaned to the left side (*Fig. S13/b*). Only a single piece of ochre was found as grave good next to the left shoulder. Three post-holes (4.5 cm in diameter) were observed in the corners of the grave-pit, which led the archaeologist to reconstruct a temporary mat-tent serving as the 'house of the dead' above the grave-pit *(163, 170)*. This burial was followed by the second building phase of the mound. The surface of the Barcé-halom is important as a botanical refugium, because it preserves an ancient loess vegetation *(173)*.

The osteological material of the Grave 1 (preserved under No. 7839 in the Anthropological Collection of the Department of Biological Anthropology, University of Szeged, Hungary) was examined and published for the first time by A. Marcsik in 1979, which analysis neatly meets ours *(174)*.

The absolute age, determined on the basis of radiocarbon dating of the human bone, is (DeA-8221) 4279±22 BP = 2916-2881 calBCE at 2σ (95.4% probability).

**SM 5 Methods CT Scan**

*Marta Petruneac, Marin Focşăneanu, Martin Trautmann*

The objects were analyzed in the Department of Applied Nuclear Physics from Horia Hulubei National Institute for R&D in Physics and Nuclear Engineering (IFIN-HH) (Romania) using a computerized tomography system. The versatile XTH225 system from Nikon offers high image resolution due to the microfocus X-ray source with 3 µm focal spot size and high performance image acquisition along with ultrafast volume processing thanks to the powerful XT software. All these great features together with a built-in 5 axes positioning system and a flat-panel detector can reveal the internal structure in precise detail.
The CT scanning of the objects were done using the following parameters: 190 kV voltage with a current intensity of 90 µA using a filter of 0.825 mm thickness of copper combined with aluminum. For each scan, there were acquired 360 projections with 4 frames per projection for a more precise image. The obtained data are visualized in Volume Graphics Studio 3.0, a very powerful image rendering software that allows us to see the inner objects as a 3D image as well as in sagittal, frontal and transverse bi-dimensional sections.

CTs were intended to support the macroscopic and visual diagnosis of two relevant features of the Strejnicu I/3 individual. First, the images of the Os sacrum should provide insight if the deviation observed in the Processus spinosus I is of traumatic origin or possibly caused by congenital deformation or by a collapse of bone tissue caused by infection (e.g. tbc), a cyst or a neoplasm. The images taken from the right femur were intended to provide information about composition and thickness of the dense cortical plaque located on the collum femoris as well as about the shape and wall thickness of the upper femoral diaphysis by non-destructive means.

## SM 6 Provenance statement

The human remains used in this study were discovered during official, systematic or rescue archaeological excavations, complying with the heritage legislation and codes of deontology of archaeological practice in the countries of Romania, Bulgaria, Hungary and Czech Republic. The researchers leading the excavations and curators of the bio-anthropological individuals on which this study is focused are included in the authors line-up. Human remains authenticity and dating were proven by archaeological context, burial customs and funerary equipment. In addition, radiocarbon dates were obtained whenever possible and are published in this study in *Table 2*. Human remains are currently stored in museums and state collections in the four countries involved.

**Supplementary figures**

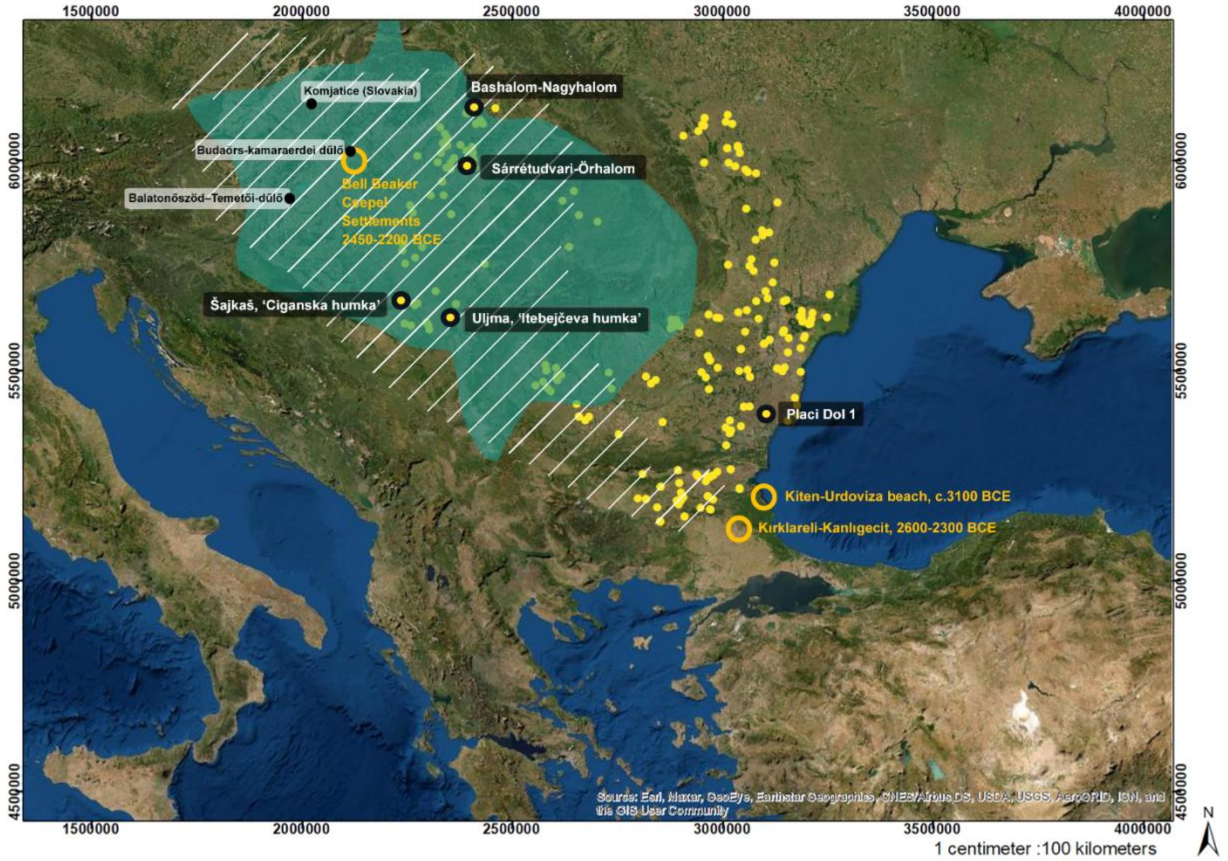


**Fig. S1. Distribution of ~3500–2500 BCE horse remain finds in east-central and southeastern Europe (background map with all excavated Yamnaya kurgans in Romania, Bulgaria, Hungary and Serbia *(15)*)**. Highlighted in black rectangles are horse remains from Yamnaya kurgans; highlighted in white rectangles are (near)complete horse skeleton finds; yellow empty circles mark settlements with significant numbers of horse bones from this period. Shaded in green is the approximate distribution of Boleráz, Baden and Coțofeni culture settlements; hatched in white is the main area in which horse remains –from some 25 sites—are currently known.

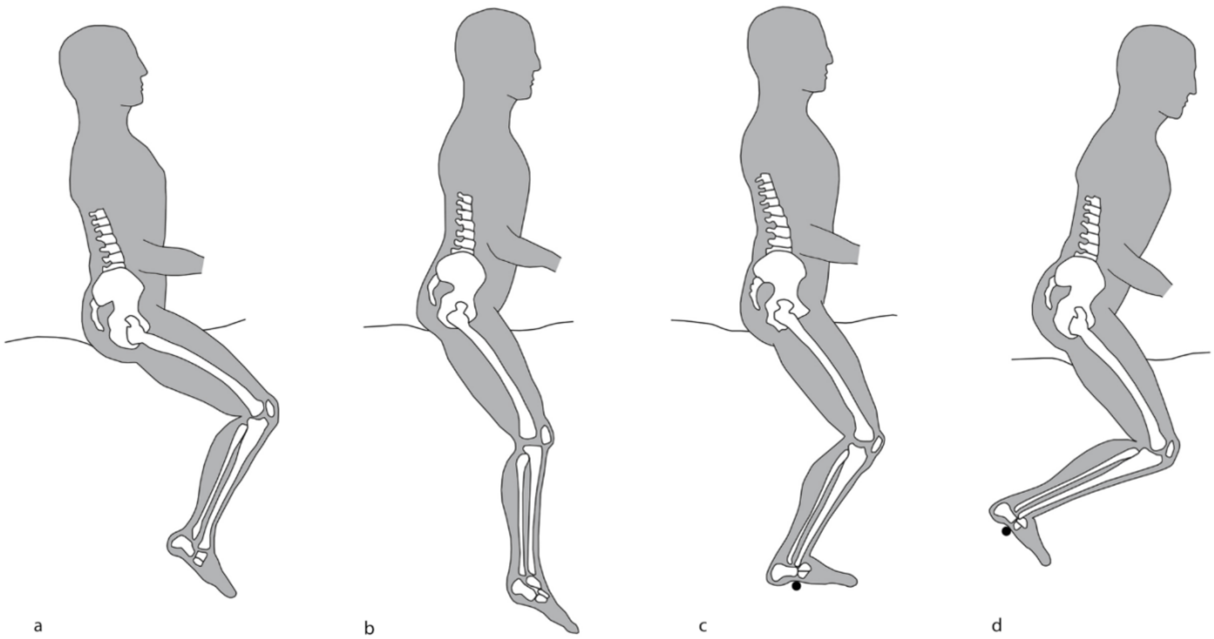


**Fig. S2. Posture in different riding styles.** a) ‘chair seat’; b) ‘split seat’; c) modern dressage seat with stirrups; d) ‘hunt seat’, slightly standing in the stirrups.

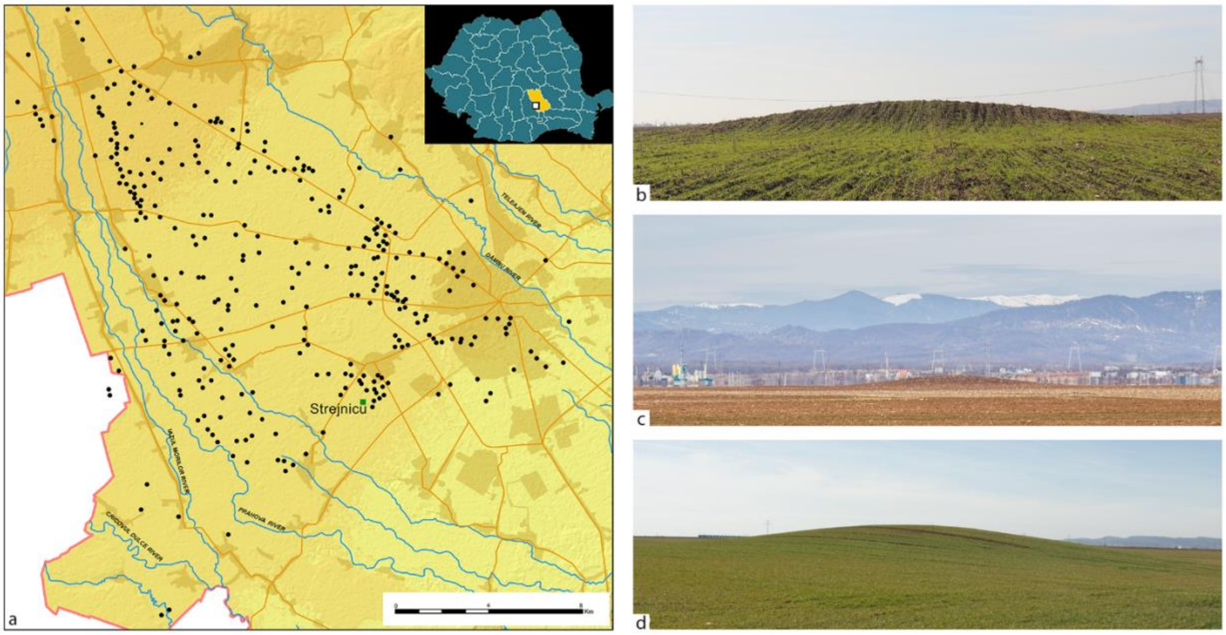


**Fig. S3. Burial mounds in Prahova region.** a. map of the mounds identified in Prahova area (Romania), on which the location of the Strejnicu mound is marked; b. photo of a mound in the Prahova area (Photo Credit: A. Frînculeasa; Prahova County Museum of History and Archaeology); c. photo of a mound in the Prahova area with the Carpathian Mountains in the background (Photo Credit: B. Preda-Bălănică; University of Helsinki); d. photo of a mound in the Prahova area (Photo Credit: A. Frînculeasa; Prahova County Museum of History and Archaeology).

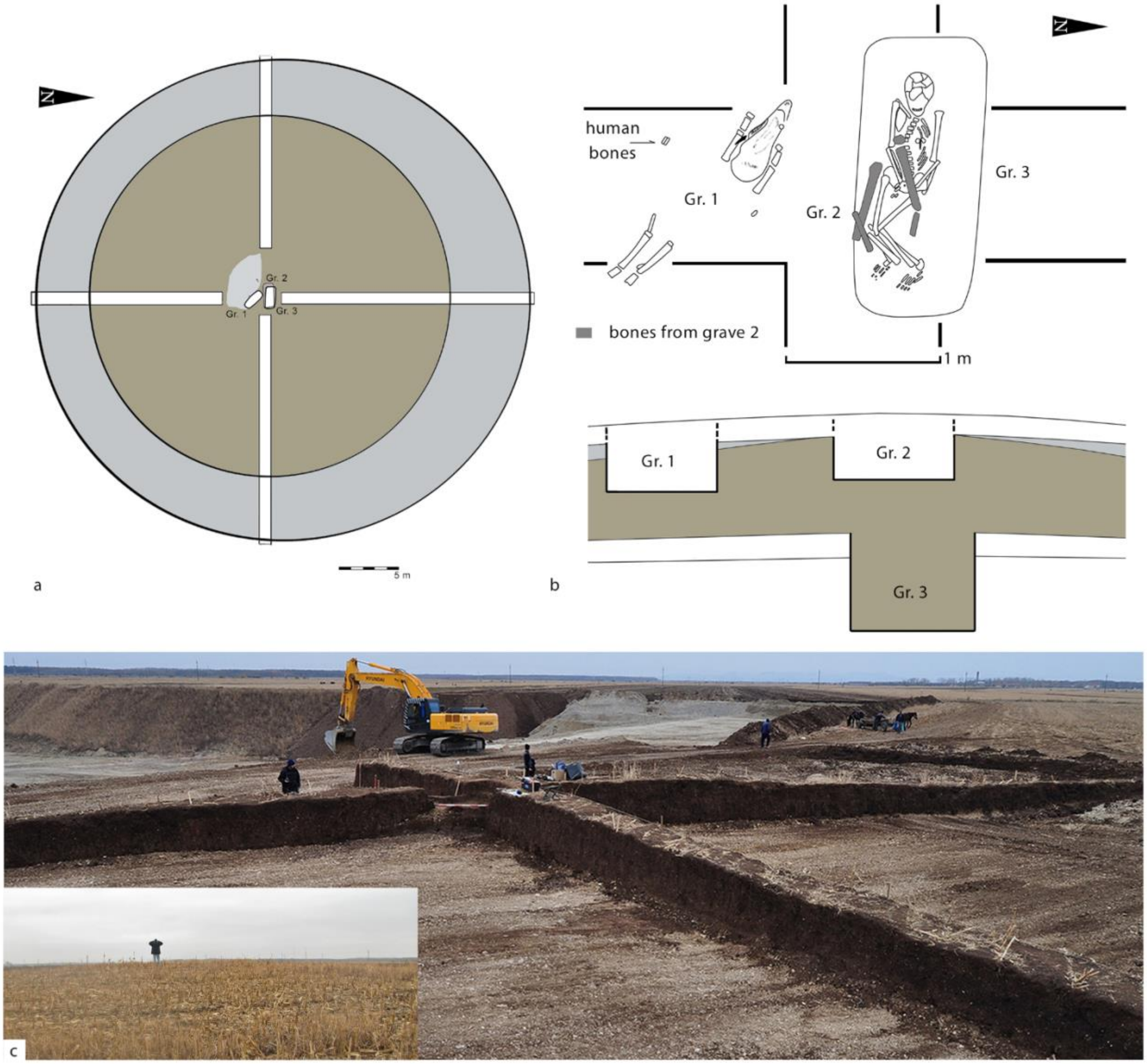


**Fig. S4. The burial mound from Strejnicu.** a. general plan of the mound from Strejnicu and the location of the three excavated burials; b. detail of the central area of the kurgan with the location of the three burials and proposed reconstruction of the profiles of grave pits; c. photos of the mound from Strejnicu before and during archaeological excavation (Photo Credit: A. Frînculeasa; Prahova County Museum of History and Archaeology) *(27)*.

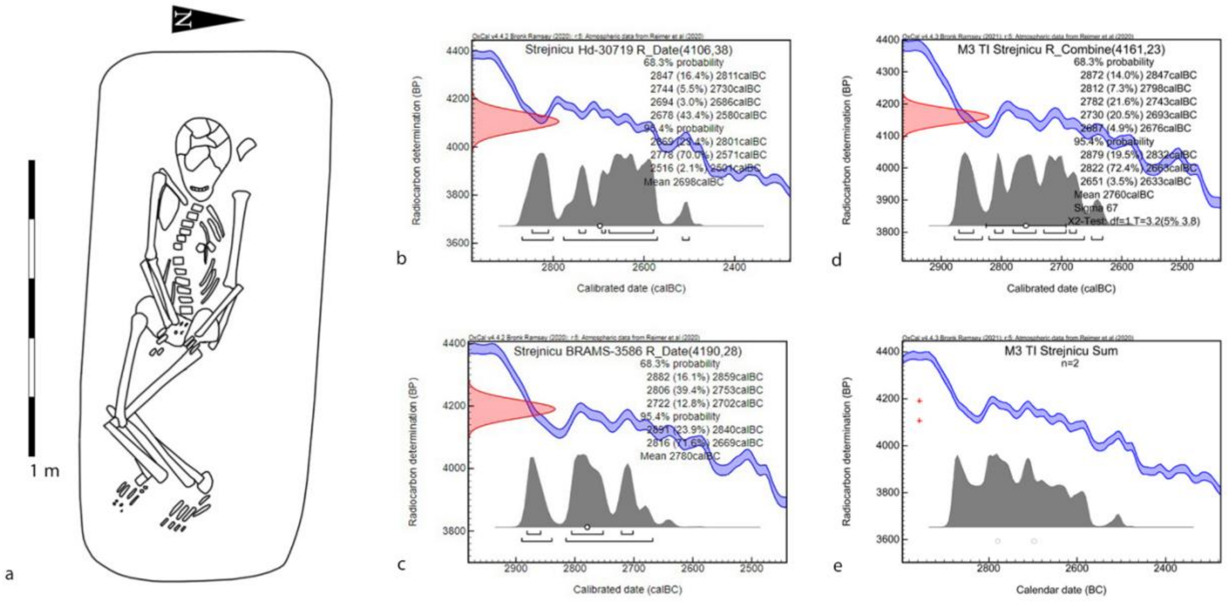


**Fig. S5. Strejnicu I/3.** a. drawing of Strejnicu I/3 Grave; b. $^{14}$C date of the Strejnicu I/3 Grave made in Heidelberg; c. $^{14}$C date of the Strejnicu I/3 Grave made in Bristol; d. R-combined value of the $^{14}$C dates of the Strejnicu I/3 Grave; e. sum of $^{14}$C dates of the Strejnicu I/3 Grave.

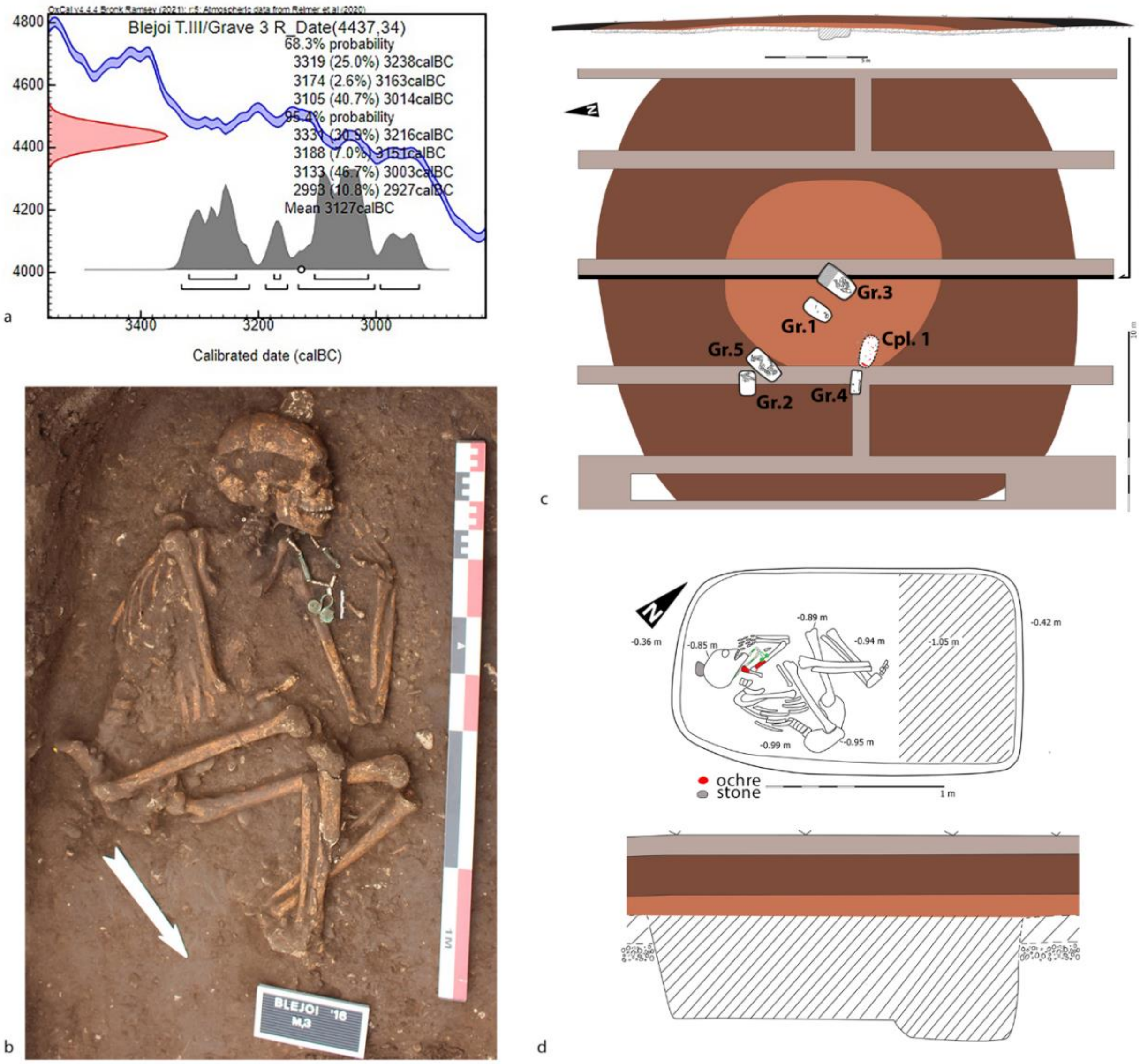


**Fig. S6. Blejoi, III/3.** a. $^{14}$C date of the Blejoi III/3 Grave; b. detail of the individual; c. general plan of the excavation; d. drawing of the grave and profile of the grave pit (Photo Credit: A. Frînculeasa; Prahova County Museum of History and Archaeology).

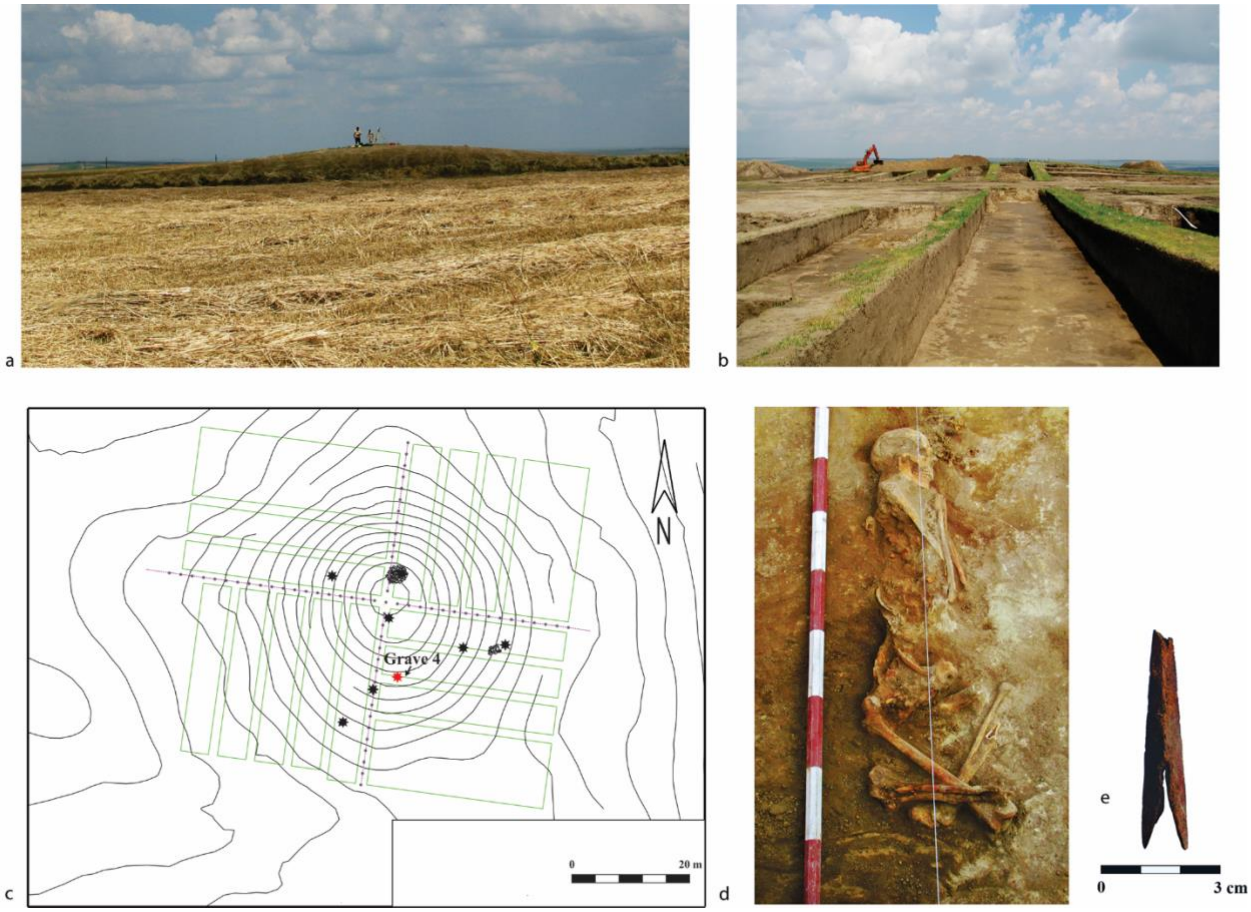


**Fig. S7. Medgidia, V/4.** a. view from SW; b. view from Medgidia mound VI, during the excavations; c. general plan of mound V; d. photo of Grave 4; e. bone arrowhead from Grave 4 (Photo Credit: A.-D. Popescu; Vasile Pârvan Institute of Archaeology).

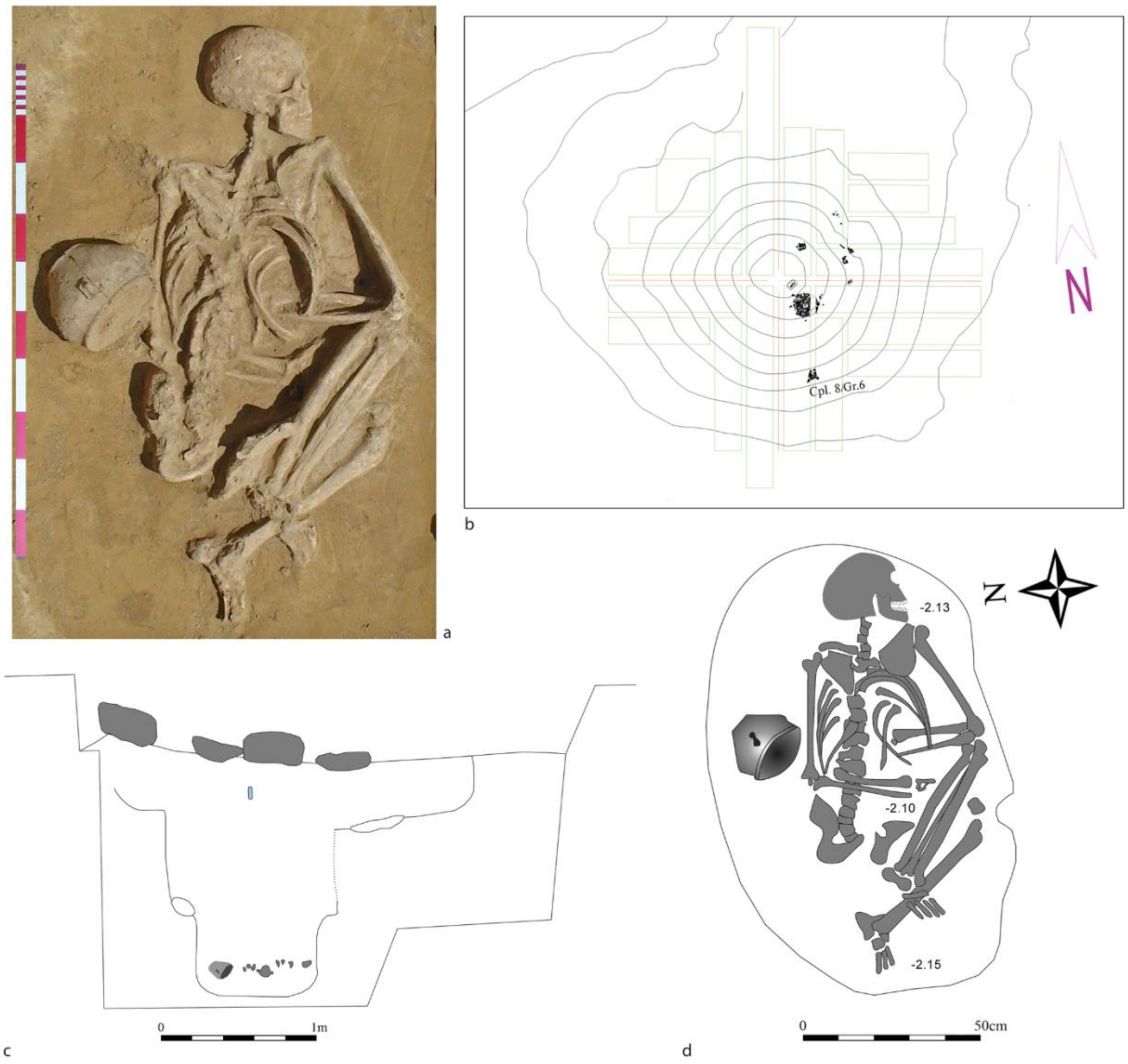


**Fig. S8. Medgidia, VI/6.** a. photo of Grave 6; b. general plan of the excavation; c. profile of the grave pit; d. drawing of Grave 6 (Photo Credit: A. Măgureanu; Vasile Pârvan Institute of Archaeology).

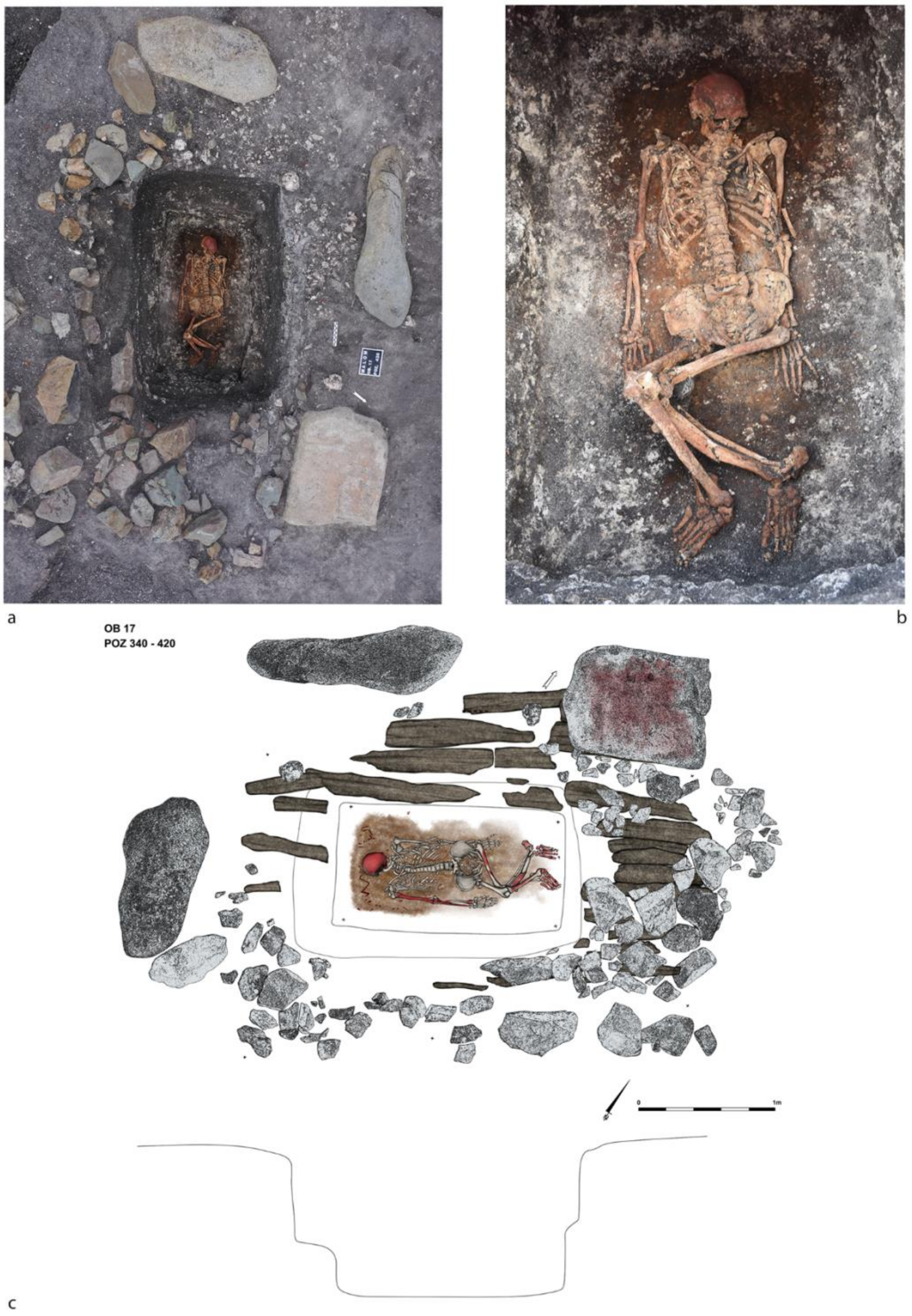


**Fig. S9. Malomirovo, Grave 17.** a. drawing of Malomirovo, Grave 17; b. photo of Malomirovo, Grave 17; c. detail of the individual in Malomirovo, Grave 17 (Photo Credit: Michał Podsiadło, Dolmen S.C.).

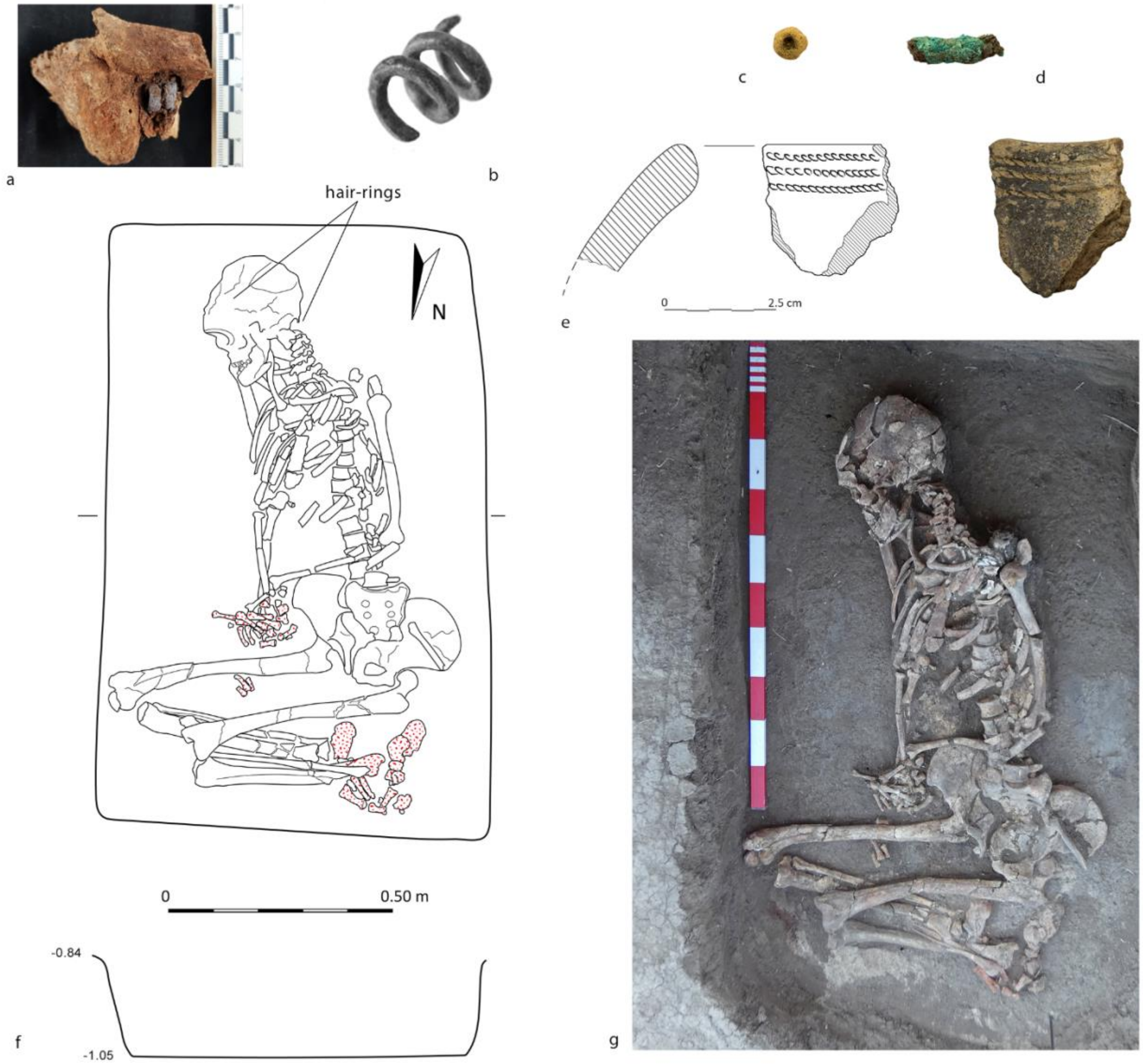


**Fig. S10. Vetrino, necropole 1, XXXIV/3.** a-b. silver hair rings; c. bone bead; d. fragment of copper/bronze wire; e. drawing and photo of cord-decorated sherd; f-g. drawing and photo of Grave 3 *(161)* (Photo Credit: S. Alexandrov, National Archaeological Institute with Museum).

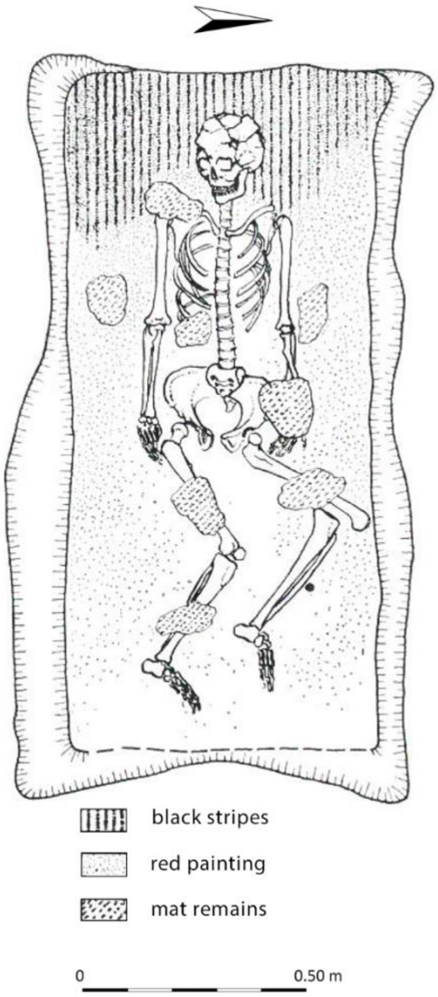


**Fig. S11. Balmazújváros – Kettőshalom, drawing of Grave 1 *(163)*.**

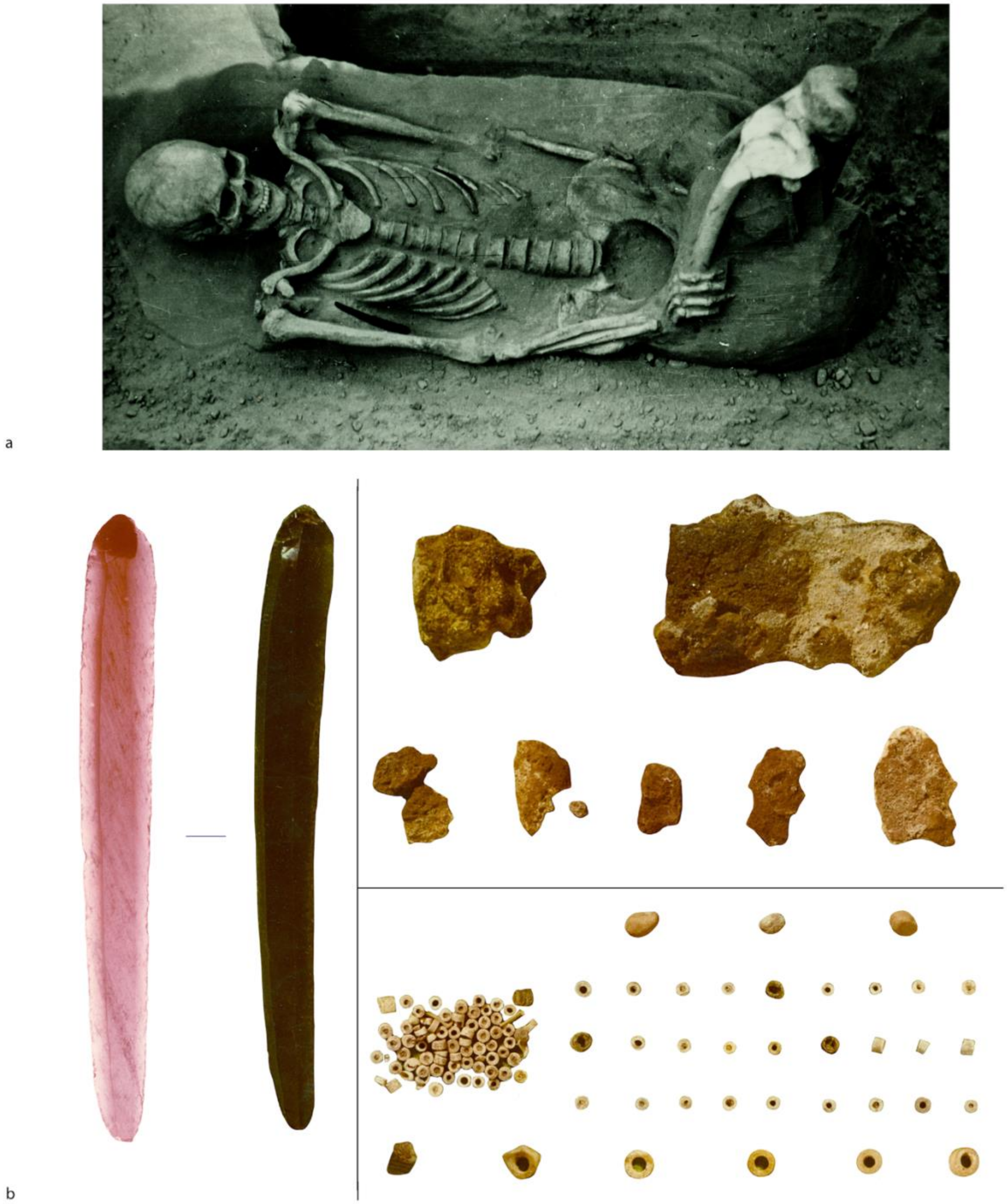

**Fig. S12. Csongrád-Kettőshalom, Bárdos-farmstead.** a. photo of Grave 1; b. grave goods *(168)* (Photo Credit: Katalin Nagy; Csongrád).

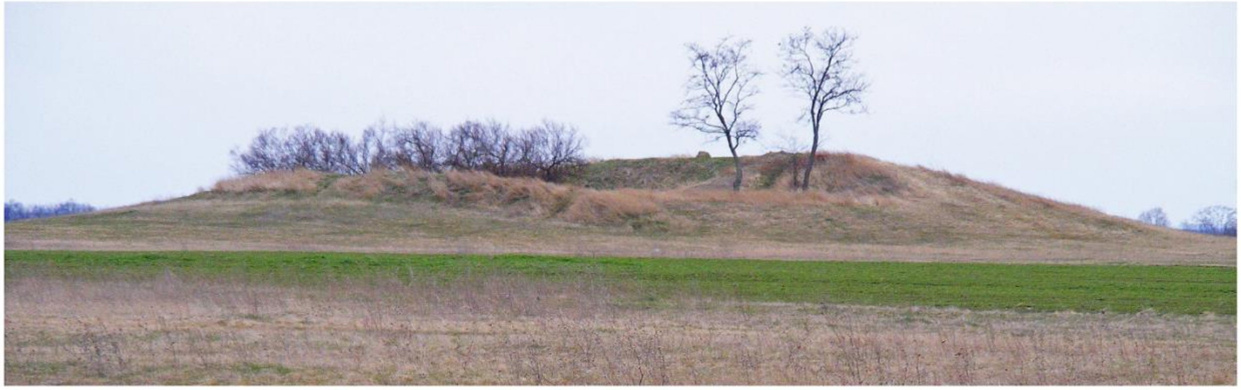

a

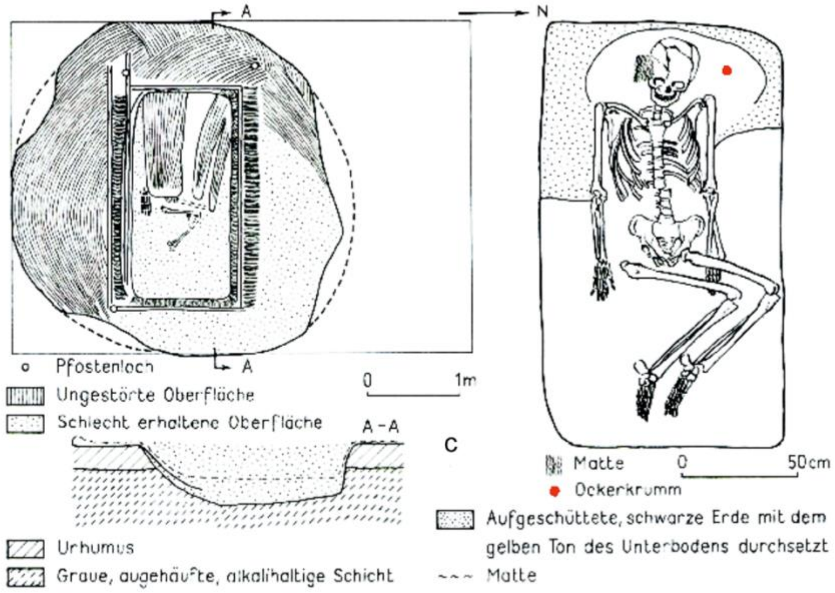


b

**Fig. S13. Dévaványa, Barcé-halom, Grave 1.** a. the disturbed body of the Barcé-halom in 2009 *(172)* (Photo Credit: Adam Bede; Lendület' Seed Ecology Research Group, Institute of Ecology and Botany); b. drawing of grave 1 *(170)*.

## REFERENCES AND NOTES

1. P. Librado, N. Khan, A. Fages, M. A. Kusliy, T. Suchan, L. Tonasso-Calvière, S. Schiavinato, D. Alioglu, A. Fromentier, A. Perdereau, J.-M. Aury, C. Gaunitz, L. Chauvey, A. Seguin-Orlando, C. Der Sarkissian, J. Southon, B. Shapiro, A. A. Tishkin, A. A. Kovalev, S. Alquraishi, A. H. Alfarhan, K. A. S. Al-Rasheid, T. Seregély, L. Klassen, R. Iversen, O. Bignon-Lau, P. Bodu, M. Olive, J.-C. Castel, M. Boudadi-Maligne, N. Alvarez, M. Germonpré, M. Moskal-del Hoyo, J. Wilczyński, S. Pospuła, A. Lasota-Kuś, K. Tunia, M. Nowak, E. Rannamäe, U. Saarma, G. Boeskorov, L. Lõugas, R. Kyselý, L. Peške, A. Bălășescu, V. Dumitrașcu, R. Dobrescu, D. Gerber, V. Kiss, A. Szécsényi-Nagy, B. G. Mende, Z. Gallina, K. Somogyi, G. Kulcsár, E. Gál, R. Bendrey, M. E. Allentoft, G. Sîrbu, V. Dergachev, H. Shephard, N. Tomadini, S. Grouard, A. Kasparov, A. E. Basilyan, M. A. Anisimov, P. A. Nikolskiy, E. Y. Pavlova, V. Pitulko, G. Brem, B. Wallner, C. Schwall, M. Keller, K. Kitagawa, A. N. Bessudnov, A. Bessudnov, W. Taylor, J. Magail, J.-O. Gantulga, J. Bayarsaikhan, D. Erdenebaatar, K. Tabaldiev, E. Mijiddorj, B. Boldgiv, T. Tsagaan, M. Pruvost, S. Olsen, C. A. Makarewicz, S. Valenzuela Lamas, S. Albizuri Canadell, A. Nieto Espinet, M. Pilar Iborra, J. Lira Garrido, E. Rodríguez González, S. Celestino, C. Olària, J. L. Arsuaga, N. Kotova, A. Pryor, P. Crabtree, R. Zhumatayev, A. Toleubaev, N. L. Morgunova, T. Kuznetsova, D. Lordkipanize, M. Marzullo, O. Prato, G. Bagnasco Gianni, U. Tecchiati, B. Clavel, S. Lepetz, H. Davoudi, M. Mashkour, N. Y. Berezina, P. W. Stockhammer, J. Krause, W. Haak, A. Morales-Muñiz, N. Benecke, M. Hofreiter, A. Ludwig, A. S. Graphodatsky, J. Peters, K. Y. Kiryushin, T.-O. Iderkhangai, N. A. Bokovenko, S. K. Vasiliev, N. N. Seregin, K. V. Chugunov, N. A. Plasteeva, G. F. Baryshnikov, E. Petrova, M. Sablin, E. Ananyevskaya, A. Logvin, I. Shevnina, V. Logvin, S. Kalieva, V. Loman, I. Kukushkin, I. Merz, V. Merz, S. Sakenov, V. Varfolomeyev, E. Usmanova, V. Zaibert, B. Arbuckle, A. B. Belinskiy, A. Kalmykov, S. Reinhold, S. Hansen, A. I. Yudin, A. A. Vybornov, A. Epimakhov, N. S. Berezina, N. Roslyakova, P. A. Kosintsev, P. F. Kuznetsov, D. Anthony, G. J. Kroonen, K. Kristiansen, P. Wincker, A. Outram, L. Orlando, The origins and spread of domestic horses from the Western Eurasian steppes. *Nature* **598**, 634–640 (2021).

2. S. Guimaraes, B. S. Arbuckle, J. Peters, S. E. Adcock, H. Buitenhuis, H. Chazin, N. Manaseryan, H.-P. Uerpmann, T. Grange, E.-M. Geigl, Ancient DNA shows domestic horses were introduced in the southern Caucasus and Anatolia during the Bronze Age. *Sci. Adv.* **6**, eabb0030 (2020).

3. S. Lindner, Chariots in the Eurasian Steppe: A Bayesian approach to the emergence of horse-drawn transport in the early second millennium BC. *Antiquity* **94** (374), 361–380 (2020).

4. D. Anthony, D. Brown, The secondary products revolution, horse-riding, and mounted warfare. *J. World Prehist.* **24**, 131 (2011).

5. W. T.-T. Taylor, C. I. Barrón-Ortiz, Rethinking the evidence for early horse domestication at Botai. *Sci. Rep.* **11**, 7440 (2021).

6. A. K. Outram, R. Bendrey, R. P. Evershed, L. Orlando, V. F. Zaibert, "Rebuttal of Taylor and Barrón-Ortiz 2021: Rethinking the evidence for early horse domestication at Botai" (2021). 10.5281/zenodo.5142604 [accessed 1 November 2022].

7. A. K. Outram, N. A. Stear, R. Bendrey, S. Olsen, A. Kasparov, V. Zaibert, N. Thorpe, R. P. Evershed, The earliest horse harnessing and milking. *Science* **323**, 1332–1335 (2009).

8. A. Outram, A. Polyakov, A. Gromov, V. Moiseyev, A. W. Weber, V. I. Bazaliiskii, O. I. Goriunova, "Archaeological supplement B to Damgaard et al. 2018: Discussion of the archaeology of Central Asian and East Asian Neolithic to Bronze Age hunter-gatherers and early pastoralists, including consideration of horse domestication" (2018). 10.5281/zenodo.1240521 [accessed 19 June 2022].

9. S. Wilkin, A. Ventresca Miller, R. Fernandes, R. Spengler, W. T.-T. Taylor, D. R. Brown, D. Reich, D. J. Kennett, B. J. Culleton, L. Kunz, C. Fortes, A. Kitova, P. Kuznetsov, A. Epimakhov, V. F. Zaibert, A. K. Outram, E. Kitov, A. Khokhlov, D. Anthony, N. Boivin, Dairying enabled Early Bronze Age Yamnaya steppe expansions. *Nature* **598**, 629–633 (2021).

10. A. Bennett, J. Weber, W. Bendhafer, S. Champlot, J. Peters, G. M. Schwartz, T. Grange, E.-M. Geigl, The genetic identity of the earliest human-made hybrid animals, the kungas of Syro-Mesopotamia. *Sci. Adv.* **8**, eabm0218 (2022).

11. D. Bodi, The mustering of tribes for battle in 1 Samuel 11 and in *Arm* II 48 and the donkey as the Hebrew royal symbol in light of Amorite customs. *Revue Int. d'Histoire Militaire Ancienne* **5**, 7–31 (2017).

12. R. Kyselý, L. Peške, Horse size and domestication: Early equid bones from the Czech Republic in the European context. *Anthropozoologica* **51**, 1–39 (2016).

13. S. Burmeister, Early wagons in Eurasia: Disentangling an enigmatic innovation, in *Appropriating Innovations Entangled Knowledge in Eurasia, 5000–1500 BCE*, P. W. Stockhammer, J. Maran, Eds. (Oxbow, 2017), pp. 69–77.

14. W. Honeychurch, L. Rogers, C. Amartuvshin, E. Diimaajav, N.-O. Erdene-Ochir, M. E. Hall, M. Hrivnyak, The earliest herders of East Asia: Examining Afanasievo entry to Central Mongolia. *Arch. Res. in Asia* **26** 100264 (2021).

15. B. Preda-Bălănică, A. Frînculeasa, V. Heyd, The Yamnaya impact North of the Lower Danube: A tale of newcomers and locals. *Bull. Soc. Préhist. française* **117**, 85–101 (2020).

16. E. Kaiser, *Das dritte Jahrtausend im osteuropäischen Steppenraum* (Berlin Studies of the Ancient World, Edition Topoi, 2019), vol. 37.

17. S. Bőkőnyi, The earliest waves of domestic horses in East Europe. *J. Indo Eur. Stud.* **6**, 17–76 (1978).

18. H. M. Clayton, S.-J. Hobbs, The role of biomechanical analysis of horse and rider in equitation science. *Appl. Anim. Behav. Sci.* **190**, 123–132 (2017).

19. G. Pálfi, O. Dutour, Activity-induced skeletal markers in historical anthropological material. *Int. J. Anthropol.* **11**, 41–55 (1996).

20. R. Jurmain, F. Alves Cardoso, C. Henderson, S. Villotte, Bioarchaeology's holy grail: The reconstruction of activity, in *A Companion to Paleopathology*, A. L. Grauer, Ed. (Wiley/Blackwell, 2012), pp. 531–542.

21. C. S. Larsen, *Bioarchaeology: Interpreting Behavior from the Human Skeleton* (Cambridge Univ. Press, ed. 2, 2015).

22. W. Berthon, "Bioarchaeological analysis of the mounted archers from the Hungarian Conquest period (10th century): Horse riding and activity-related skeletal changes," thesis, University of Szeged, Hungary and École Pratique des Hautes Études, PSL University, Paris, France (2019).

23. E. Bagagli, F. Cantini, F. Mallegni, F. Bartoli, "Horseman Syndrome" in the Tuscan Early Middle Age: The Sk888 case. *J. Biol. Res.* **85**, 203–204 (2012).

24. J. Zaia, *Saddle Sore: Skeletal Occupational Markers of Habitual Horseback Riding* (Senior Honors Theses 269, Brockport NY, 2019); http://hdl.handle.net/20.500.12648/6765

25. K. Djukic, N. Miladinovic-Radmilovic, M. Draskovic, M. Djuric, Morphological appearance of muscle attachment sites on lower limbs: Horse riders versus agricultural population. *Int. J. Osteoarchaeol.* **28**, 656–668 (2018).

26. G. Pálfi, Traces des activités sur les squelettes des anciens Hongrois. *Bull. et Mémoires Soc. d'Anthropologie de Paris* **4**, 209–231 (1992).

27. A. Frînculeasa, B. Preda, V. Heyd, Pit-graves, Yamnaya and Kurgans along the Lower Danube: Disentangling 4$^{th}$ and 3$^{rd}$ millennium BC burial customs, equipment and chronology. *Praehist. Zeitschrift* **90**, 45–113 (2015).

28. S. Villotte, C. J. Knüsel, Understanding entheseal changes: Definition and life course changes. *Int. J. Osteoarchaeol.* **23**, 135–146 (2013).

29. S. Stefanović, M. Porčić, Between-group differences in the patterning of musculo-skeletal stress markers: Avoiding confounding factors by focusing on qualitative aspects of physical activity. *Int. J. Osteoarchaeol.* **23**, 94–105 (2013).

30. J. D. Erickson, D. V. Lee, J. E. A. Bertram, Fourier analysis of acetabular shape in Native American Arikara populations before and after acquisition of horses. *Am. J. Physical Anthropol.* **113**, 473–480 (2000).

31. W. Berthon, B. Tihanyi, L. Kis, L. Révész, H. Coqueugniot, O. Dutour, G. Pálfi, Horse riding and the shape of the acetabulum: Insights from the bioarchaeological analysis of early Hungarian mounted archers (10th century). *Int. J. Osteoarchaeol.* **29**, 117–126 (2019).

32. S. Villotte, C. J. Knüsel, Some remarks about femoroactebular impingement and osseous non-metric variations of the proximal femur. *Bull. et Mémoires Soc. d'Anthropologie de Paris* **21**, 95–98 (2009).

33. N. Radi, V. Mariotti, A. Riga, S. Zampetti, C. Villa, M. G. Belcastro, Variation of the anterior aspect of the femoral head-neck junction in modern human identified skeletal collection. *Am. J. Phys. Anthropol.* **152**, 261–272 (2013).

34. C. J. Knüsel, Bone adaptation and its relationship to physical activity in the past, in *Human Osteology in Archaeology and Forensic Science*, M. Cox, S. Mays, Eds. (Greenwich Medical Media Ltd. and Cambridge Univ. Press, 2000), pp. 381–402.

35. D. J. Wescott, "Structural variation in the humerus and femur in the American Great Plains and adjacent regions: Differences in subsistence strategy and physical terrain," thesis, The University of Tennessee, Knoxville, TN (2001).

36. B. K. McIlvaine, L. A. Schepartz, Femoral subtrochanteric shape variation in Albania: Implications for use in forensic applications. *Homo* **66**, 79–89 (2015).

37. C. Shaw, J. Stock, Intensity, repetitiveness, and directionality of habitual adolescent mobility patterns influence the tibial diaphysis morphology of athletes. *Am. J. Phys. Anthropol.* **140**, 149–159 (2009).

38. C. N. Kraft, P. H. Pennekamp, U. Becker, M. Young, O. Diedrich, C. Lüring, M. von Falkenhausen, Magnetic resonance imaging findings of the lumbar spine in elite horseback riders: Correlations with back pain, body mass index, trunk/leg-length coefficient, and riding discipline. *Am. J. Sports Med.* **37**, 2205–2213 (2009).

39. K. D. Williams, N. J. Meinzer, C. S. Larsen, History of degenerative `joint disease in people across Europe, in *The Backbone of Europe Health, Diet, Work and Violence over Two Millennia*, R. H. Steckel, C. S. Larsen, C. A. Roberts, J. Baten, Eds. (Cambridge Univ. Press, 2018), pp. 253–299.

40. R. T. Loder, The demographics of equestrian-related injuries in the United States: Injury patterns, orthopedic specific injuries, and avenues for injury prevention. *J. Trauma* **65**, 447–460 (2008).

41. W. Berthon, B. Tihanyi, O. A. Váradi, H. Coqueugniot, O. Dutour, G. Pálfi, Riding for a fall: Bone fractures among mounted archers from the Hungarian Conquest period (10th century CE). *Int. J. Osteoarchaeol.* **31**, 926–940 (2021).

42. D. Anthony, *The Horse, the Wheel, and Language: How Bronze-Age Riders from the Eurasian Steppes Shaped the Modern World* (Princeton Univ. Press, 2007).

43. K. Kanne, Riding, ruling, and resistance: Equestrianism and political authority in the Hungarian Bronze Age. *Curr. Anthropol.* **63**, 289–329 (2022).

44. T. Molleson, J. Blondiaux, Riders' bones from Kish, Iraq. *Cambridge Archaeol. J.* **4**, 312–316 (1994).

45. A. Khazanov, *Nomads and the Outside World* (Cambridge Univ. Press, 1984).

46. D. H. Ubelaker, *Human Skeletal Remains: Excavation, Analysis, Interpretation* (Taraxacum, 1978).

47. D. R. Brothwell, *Digging Up Bones, The Excavation, Treatment and Study of Human Skeletal Remains* (Cornell Univ. Press, ed. 3, 1981).

48. J. E. Buikstra, D. H. Ubelaker, *Standards for Data Collection from Human Skeletal Remains. Proceedings of a Seminar at the Field Museum of Natural History* (Arkansas Archaeological Survey Research Series 44, 1994).

49. A. Chamberlain, *Demography in Archaeology* (Cambridge Manuals in Archaeology, Cambridge Univ. Press, 2006).

50. R. H. Steckel, C. S. Larsen, P. W. Sciulli, P. L. Walker, *The Global History of Health Project. Data Collection Codebook* (2006); http://global.sbs.ohio-state.edu/docs/Codebook-12-12-05.pdf.

51. G. Grupe, M. Harbeck, G. McGlynn, *Prähistorische Anthropologie* (Springer-Verlag Berlin Heidelberg, 2015).

52. W. W. Howells, "Who's who in skulls. Ethnic identification of crania from measurements" (Papers of the Peabody Museum of Archaeology and Ethnology 82, Peabody Museum, 1995).

53. W. W. Howells, "Cranial variation in man. A study by multivariate analysis of patterns of differences among recent human populations" (Papers of the Peabody Museum of Archeology and Ethnology 67, Peabody Museum, 1973).

54. E. Cunha, C. Umbelino, What can bones tell about labour and occupation: The analysis of skeletal markers of occupational stress in the Identified Skeletal Collection of the Anthropological Museum of the University of Coimbra (preliminary results). *Antropol. Port.* **13**, 49–68 (1995).

55. C. S. Larsen, P. L. Walker, R. H. Steckel, P. Sciulli, H. D. Klaus, J. Blondiaux, G. Grupe, R. Jankauskas, G. Maat, G. McGlynn, A. Papathanasiou, C. Roberts, M. Teschler-Nicola, U. Wittwer-Backofen, A. Agnew, S. Assis, Z. Bereczki, B. Bertrand, T. K. Betsinger, S. Boulter, C. Bourbou, A. Boylston, M. Brickley, L. Bürli, C. Cooper, A. Coppa, J. Coughlan, A. Drozd, E. During, J. Eng, F. Engel, S. Fox, M. Furtado, G. Gerhards, K. Haebler, K. Harkins, P. Holck, M. Holst, G. Hotz, H. Justus, K. Kaminska, A. Kjellström, C. J. Knüsel,

T. Kozlowski, A. Lagia, C. Lopes, S. Manolis, A. Marcsik, C. Marques, C. Moenke, I. Moutafi, C. Niel, S. A. Novak, F. Novotny, J. Peck, I. Potiekhina, B. Rega, R. Richman, F. Rijpma, J. Rose, J. Ruiz, P. Sannen, A. Soficaru, M. Spannagl, R. Storm, M. E. Subirà, D. Swales, V. Tritsaroli, E. Tyler, S. Ulrich-Bochsler, S. Vatteoni, N. Villena-Mota, R. Wiggins, L. L. Williams, *History of Degenerative Joint Disease in Europe: Inferences about Lifestyle and Activity* (AAPA Symposium Reconstructing Health and Disease in Europe: The Early Middle Ages through the Industrial Period, 2009).

56. S. Villotte, S. Assis, F. Alves Cardoso, C. Y. Henderson, V. Mariotti, M. Milella, D. Pany-Kucera, N. Speith, C. A. Wilczak, R. Jurmain, In search of consensus: Terminology for entheseal changes (EC). *Int. J. Paleopathol.* **13**, 49–55 (2016).

57. M. Miller, F. A. Cardoso, S. Assis, G. P. Lopreno, N. Speith, Exploring the relationship between entheseal changes and physical activity: A multivariate study. *Am. J. Phys. Anthropol.* **156**, 215–223 (2015).

58. A. Azzopardi, D. W. Bartlett, T. F. Watson, B. G. Smith, A literature review of the techniques to measure tooth wear and erosion. *Eur. J. Prosthodont. Restor. Dent.* **8**, 93–97 (2000).

59. K. W. Alt, F. W. Rösing, M. Teschler-Nicola, *Dental Anthropology, Fundamentals, Limits and Prospects* | Softcover reprint of the original 1st ed. 1998 (Springer, 2011).

60. C. Ganss, J. Klimek, N. Borkowski, Characteristics of tooth wear in relation to different nutritional patterns including contemporary and medieval subjects. *Eur. J. Oral Sci.* **110**, 54–60 (2002).

61. H. Schutkowski, Thoughts for food: Evidence and meaning of past dietary habits, in *Between Biology and Culture* (Cambridge Studies in Biological and Evolutionary Anthropology 56, 2008), pp. 141–164.

62. B. H. Smith, Patterns of molar wear in hunter-gatherers and agriculturalists. *Am. J. Phys. Anthropol.* **63**, 39–56 (1984).

63. A. C. Aufderheide, C. Rodríguez-Martín, *The Cambridge Encyclopedia of Human Paleopathology* (Cambridge Univ. Press, 1998).

64. A. L. Grauer, Macroscopic analysis and data collection in palaeopathology, in *Advances in Human Palaeopathology,* R. Pinhasi, S. Mays, Eds. (Wiley, 2008), pp. 57–76.

65. M. N. Cohen, G. J. Armelagos, *Paleopathology at the Origins of Agriculture: Proceedings of the Conference on Paleopathology and Socioeconomic Change at the Origins of Agriculture at Plattsburgh, held April 25–May 1, 1982* (Academic Press, 1984).

66. D. J. Ortner, *Identification of Pathological Conditions in Human Skeletal Remains* (Elsevier, 2003).

67. C. A. Roberts, Adaptation of populations to changing environments: Bioarchaeological perspectives on health for the past, present and future. *Bull. Mém. Soc. Anthropol. Paris* **22**, 38–46 (2010).

68. K. Alt, *Odontologische Verwandtschaftsanalyse: Individuelle Charakteristika der Zähne in ihrer Bedeutung für Anthropologie, Archäologie und Rechtsmedizin* (Fischer Verlag GmbH & Co. KG, 1998).

69. R. W. Mann, D. R. Hunt, S. Lozanoff, *Photographic Regional Atlas of Non-Metric Traits and Anatomical Variants in the Human Skeleton* (Charles C Thomas, 2016).

70. M. Trautmann, Die schnurkeramischen Bestattungen von Lauda-Königshofen. Steinzeitliche Hirtennomaden im Taubertal? *Fundberichte aus Baden-Württemberg* **32**, 265–476 (2012).

71. J. E. Buikstra, L. A. Beck, *Bioarchaeology: The Contextual Analysis of Human Remains* (Academic Press, 2006).

72. D. J. Wescott, Effects of mobility on femur midshaft external shape and robusticity. *Am. J. Phys. Anthropol.* **130**, 201–213 (2006).

73. C. B. Ruff, Biomechanical analysis of archaeological human skeletons, in *Biological Anthropology of the Human Skeleton*, M. A. Katzenberg, S. R. Saunders, Eds. (Wiley-Liss, 2000), pp. 71–102.

74. C. S. Larsen, Biological changes in human populations with agriculture. *Ann. Rev. Anthropol.* **24**, 185–213 (1995).

75. M. Wagner, X. Wu, P. Tarasov, A. Aisha, C. Bronk Ramsey, M. Schultz, T. Schmidt-Schultz, J. Gresky, Radiocarbon-dated archaeological record of early first millennium B.C. mounted pastoralists in the Kunlun Mountains, China. *Proc. Natl. Acad. Sci. U.S.A.* **108**, 15733–15738 (2011).

76. M. Levine, C. Renfrew, K. Boyle, *Prehistoric Steppe Adaptation and the Horse* (McDonald Institute for Archaeological Research, 2003).

77. C. Renfrew, Pastoralism and interaction: Some introductory questions, in *Ancient Interactions: East and West in Eurasia*, K. Boyle, C. Renfrew, M. Levine, Eds. (McDonald Institute for Archaeological Research, 2002), pp. 1–12.

78. E. Rosenstock, M. Groß, A. Hujić, A. Scheibner, Back to good shape: Biological standard of living in the Copper and Bronze Ages and the possible role of food, in *Human Development in Landscapes. Vol. 6. The Third Food Revolution? Setting the Bronze Age table: Common Trends in Economic and Subsistence Strategies in Bronze Age Europe. Proceedings of the International Workshop "SocioArchaeol Anthropol Sci Environmental Dynamics over the Last 12,000 Years: The Creation of Landscapes III (15th–18th April 2013)" in Kiel*, J. Kneisel, M. Dal Corso, W. Kirleis, H. Scholz, N. Taylor, V. Tiedtke, Eds. (Habelt, 2015), pp. 121–152.

79. R. Steckel, C. S. Larsen, C. A. Roberts, J. Baten, *The Backbone of Europe: Health, Diet, Work and Violence over Two Millennia* (Cambridge Univ. Press, 2018).

80. M. Niskanen, C. B. Ruff, B. Holt, V. Sládek, M. Berner, Temporal and geographic variation in body size and shape of europeans from the Late Pleistocene to recent times, in *Skeletal*

*Variation and Adaptation in Europeans: Upper Paleolithic to the Twentieth Century*, C. B. Ruff, Ed. (Wiley Blackwell, 2018), pp. 49–89.

81. D. I. Owen, The first equestrian: An Ur III glyptic scene. *Acta Sumerologica* **13**, 259–273 (1991).

82. Collection of the British Museum, Inv. No. 22958; www.britishmuseum.org/collection/object/W_1897-0511-104 [accessed 5 April 2021].

83. F. A. M. Wiggermann, The seal of Ili-Padda, grand vizier of the Middle Assyrian Empire, in *The Iconography of Cylinder Seals*, P. Taylor, Ed. (University of London Press, 2006), pp. 92–99.

84. Collection of the Ägyptisches Museum und Papyrussammlung Berlin, ÄM 21826 (Photo credit: S. Steiß, Berlin).

85. Collection of the Metropolitan Museum New York, Acc. No. 05.3.263; www.metmuseum.org/art/collection/search/548708 [accessed 30 November 2022].

86. Collection of the Museo Civico Archeologico di Bologna, inv MCABo 1899; www.museibologna.it/archeologicoen/percorsi/66288/id/74955/oggetto/74958/ [accessed 9 November 2022].

87. J. Kelder, Horseback riding and cavalry in Mycenaean Greece. *Anc. West East* **11**, 11–18 (2012).

88. J. Dani, Milleker's pride and joy, in *Danubian Route of the Yamnaya Culture: The Barrows of Vojvodina*, P. Jarosz, J. Koledin, P. Włodarczak, Eds. (The Yamnaya Impact of Prehistoric Europe 3, Archaeolingua, 2021), pp. 195–206.

89. D. Makowicz-Poliszot, Analysis of animal bones from "Ciganska humka" in ŠajkaŠ, in *Danubian Route of the Yamnaya Culture: The Barrows of Vojvodina*, P. Jarosz, J. Koledin, P. Włodarczak, Eds. (The Yamnaya Impact of Prehistoric Europe 3, Archaeolingua, 2021), pp. 91–102.

90. S. Bökönyi, Copper Age vertebrae fauna from Kétegyháza, in *The People of the Pit-grave Kurgans in Eastern Hungary*, I. Ecsedy, Eds. (Fontes Arch. Hungariae, 1979), pp. 101–116.

91. C. Gerling, E. Bánffy, J. Dani, K. Köhler, G. Kulcsár, A. W. G. Pike, V. Szeverényi, V. Heyd, Immigration and transhumance in the Early Bronze Age Carpathian Basin: The occupants of akurgan. *Antiquity* **86**, 1097–1111 (2012).

92. S. Alexandrov, Fourth/third millennium BC barrow graves in North-East Bulgaria (120 years of investigations), in *Yamnaya Interactions. Proceedings of the International Workshop held in Helsinki, 25–26 April 2019*, V. Heyd, G. Kulcsár, B. Preda-Bălănică, Eds. (The Yamnaya Impact of Prehistoric Europe 2, Archaeolingua, 2021), pp. 271–314.

93. N. Benecke, Diachroner Vergleich der Pferdehaltung im Karpatenbecken und in der osteuropäischen Steppe während der vorchristlichen Metallzeiten, in *Das Karpatenbecken und die osteuropäische Steppe*, B. Hänsel, J. Machnik, Eds. (Südosteuropa-Schriften 20, Prähistorische Archäologie Südosteuropa 12, Leidorf, 1998), pp. 91–98.

94. E. Gál, Animal bone remains from the Late Copper Age cemetery at Pilismarót-Basaharc, in *The Late Copper Age cemetery at Pilismarót-Basaharc*, M. Bondár, Ed. (Archaeolingua, 2015), pp. 367–379.

95. E. Gál, *Animals at the Dawn of Metallurgy in South-Western Hungary* (Archaeolingua, 2017).

96. N. Benecke, On the beginning of horse husbandry in the Southern Balkan peninsula—The horses from Kanlıgeçit, Kırklareli (Turkish Thrace). *Turkish Acad. Sci. J. Archaeol.* **12**, 13–23 (2009).

97. L. György, Late Copper Age Animal Burials in the Carpathian Basin, in *Moments in Time. Papers Presented to Pál Raczky on His 60th Birthday,* A. Anders, G. Kulcsár, Eds. (Prehistoric Society, Eötvös Loránd Univ., L'Harmattan, 2013), pp. 627–642.

98. N. Spassov, L. Hristova, N. Iliev, The domesticated horses from the submerged prehistoric village of Urdoviza (Kiten) on the Bulgarian Black Sea coast—Among the oldest known. *Hist. Nat. Bulga.* **25**, 11–14 (2018).

99. K. Lyublyanovics, Animal bones from the Bell Beaker Settlement of Albertfalva, Budapest, in *A Bell Beaker Settlement in Albertfalva, Hungary (2470–1950 BC)*, A. Endrödi, L. Reményi, Eds. (Budapest History Museum, 2016), pp. 204–216.

100. C. Gaunitz, A. Fages, K. Hanghøj, A. Albrechtsen, N. Khan, M. Schubert, A. Seguin-Orlando, I. J. Owens, S. Felkel, O. Bignon-Lau, P. de Barros Damgaard, A. Mittnik, A. F. Mohaseb, H. Davoudi, S. Alquraishi, A. H. Alfarhan, K. A. S. Al-Rasheid, E. Crubézy, N. Benecke, S. Olsen, D. Brown, D. Anthony, Ken Massy, V. Pitulko, A. Kasparov, G. Brem, M. Hofreiter, G. Mukhtarova, N. Baimukhanov, L. Lõugas, V. Onar, P. W. Stockhammer, J. Krause, B. Boldgiv, S. Undrakhbold, D. Erdenebaatar, S. Lepetz, M. Mashkour, A. Ludwig, B. Wallner, V. Merz, I. Merz, V. Zaibert, E. Willerslev, P. Librado, A. K. Outram, L. Orlando, Ancient genomes revisit the ancestry of domestic and Przewalski's horses. *Science* **360**, 111–114 (2018).

101. D. Y. Telegin, A. L. Nechitailo, I. D. Potekhina, Y. V. Panchenko, *Srednestogovskaya i Novodanilovskaya Kul'tury Eneolita Azovo-Chernomorskogo Regiona* (Lugansk, 2001).

102. S. Reinhold, J. Gresky, N. Berezina, A. R. Kantorovich, C. Knipper, V. E. Maslov, V. G. Petrenko, K. W. Alt, A. B. Belinsky, Contextualising innovation. Cattle owners and wagon drivers in the North Caucasus and beyond, in *Appropriating Innovations: Entangled Knowledge in Eurasia, 5000–1500 BCE*, P. Stockhammer, J. Maran, Eds. (Oxbow, 2017), pp. 78–97.

103. P. F. Kuznetsov, A. N. Usachuk, Obshchee i osobennoe v izgotovlenii psaliev iz Ureni—Detalei upryadzhi boevykh kolesnits. *Strat. Plus* **2**, 335–341 (2019).

104. D. W. Anthony, A. A. Khokhlov, S. A. Agapov, D. S. Agapov, R. Schulting. I. Olalde, D. Reich, The Eneolithic cemetery at Khvalynsk on the Volga River. *Praehistorische Zeitschrift* **97**, 22–67 (2022).

105. E. Rosenstock, A. Masson, B. Zich, Moraines, megaliths and moo: Putting the prehistoric tractor to work, in *Megaliths – Societies – Landscapes: Early Monumentality and Social Differentiation in Neolithic Europe, Volume 3, Proceedings of the International Conference »Megaliths – Societies – Landscapes. Early Monumentalityand Social Differentiation in Neolithic Europe« (16th–20th June 2015) in Kiel*, J. Müller, M. Hinz, M. Wunderlich, Eds. (Dr. Rudolf Habelt GmbH, 2019) pp. 1099–1110.

106. A. Sherratt, The secondary exploitation of animals in the Old World. *World Archaeol.* **15**, 90–104 (1983).

107. N. L. Morgunova, *Pri-Uralskaya Gruppa Pamyatnikov v Sisteme Volzhsko-Ural'skogo Varianta Yamnoi Kul'turno-Istoricheskoi Oblasti* (OGPU, 2014).

108. N. I. Shishlina, *Reconstruction of the Bronze Age of the Caspian Steppes: Life Styles and Life Ways of Pastoral Nomads* (British Archaeological Reports International Series 1876, 2008).

109. R. J. Schulting, M. P. Richards, Stable isotope analysis of neolithic to Late Bronze Age populations in the Samara Valley, in *A Bronze Age Landscape in the Russian Steppes: The Samara Valley Project*, D. W. Anthony, D. Brown, O. Mochalov, A. Khokhlov, P. Kuznetsov, Eds. (Monumenta Archaeologica 37, Cotsen Institute of Archaeology Press, 2016), pp. 127–147.

110. N. I. Shishlina, E. S. Azarov, T. D. Dyatlova, N. V. Roslyakova, O. P. Bachura, J. van der Plicht, P. I. Kalinin, I. A. Idrisov, A. V. Borisov, Innovatsioniye sezonnye i sistema zhineobespecheniya podvizhn'kh skotovodov v pustynno-stepnoi zone Evrazi: pol' sotsial'nykh grupp. *Strat. Plus* **2**, 69–90 (2018).

111. C. Knipper, S. Reinhold, J. Gresky, N. Berezina, C. Gerling, S. L. Pichler, A. P. Buzhilova, A. R. Kantorovich, V. E. Maslov, V. G. Petrenko, S. V. Lyakhov, A. A. Kalmykov, A. B. Belinskiy, S. Hansen, K. W. Alt, Diet and subsistence in Bronze Age pastoral communities from the southern Russian steppes and the North Caucasus. *PLOS ONE* **15**, e0239861 (2020).

112. A. Scott, S. Reinhold, T. Hermes, A. A. Kalmykov, A. Belinskiy, A. Buzhilova, N. Berezina, A. R. Kantorovich, V. E. Maslov, F. Guliyev, B. Lyonnet, P. Gasimov, B. Jalilov, J. Eminli, E. Iskandarov, E. Hammer, S. E. Nugent, R. Hagan, K. Majander, P. Onkamo, K. Nordqvist, N. Shishlina, E. Kaverzneva, A. I. Korolev, A. A. Khokhlov, R. V. Smolyaninov, S. V. Sharapova, R. Krause, M. Karapetian, E. Stolarczyk, J. Krause, S. Hansen, W. Haak, C. Warinner, Emergence and intensification of dairying in the Caucasus and Eurasian steppes. *Nat. Ecol. Evol.* **6**, 813–822 (2022).

113. S. V. Ivanova, O konseptsii vostochnogo poroisxhozhdeniya Yamnao kul'turno-istoricheskoi obshchnosti. *Voprosy Arkheologii Povol'zhya (Samara)* **4**, 203–208 (2006).

114. N. Y. Merpert, *Drevneishie skotovody Volzhsko-Ural'skogo Mezhdurech'ya* (Nauka, 1974).

115. V. P. Shilov, Modeli skotovodcheskikh khozyaistv stepnykh oblastei Evrazii v epokhu eneolita i rannego bronzovogo veka. *Sovietskaya Arkheologiya* **1**, 5–15 (1975).

116. D. W. Anthony, The Samara Valley Project and the evolution of pastoral economies in the western Eurasian steppes, in *A Bronze Age Landscape in the Russian Steppes: The Samara Valley Project*, D. W. Anthony, D. Brown, P. Kuznetsov, O. Mochalov, A. Khokhlov, Eds. (Monumenta Archaeologica 37, Cotsen Institute of Archaeology Press, 2016), pp. 3–36.

117. A. Bogaard, M. Fochesato, S. Bowles, The farming-inequality nexus: New insights from ancient Western Eurasia. *Antiquity* **93**, 1129–1143 (2019).

118. K. Kristiansen, *Archaeology and the Genetic Revolution in European Prehistory (Elements in the Archaeology of Europe)* (Cambridge Univ. Press, 2022).

119. H. M. Frost, Wolff's Law and bone's structural adaptations to mechanical usage: An overview for clinicians. *Angle Orthod.* **64**, 175–188 (1994).

120. M. Benjamin, T. Kumai, S. Milz, B. M. Boszczyk, A. A. Boszczyk, J. R. Ralphs, The skeletal attachment of tendons—Tendon 'entheses'. *Comp. Biochem. Physiol. A Mol. Integr. Physiol.* **133**, 931–945 (2002).

121. K. Djukic, P. Milovanovic, M. Hahn, B. Busse, M. Amling, M. Djuric, Bone microarchitecture at muscle attachment sites: The relationship between macroscopic scores of entheses and their cortical and trabecular microstructural design. *Am. J. Phys. Anthropol.* **157**, 81–93 (2015).

122. L. Capasso, K. A. R. Kennedy, C. A. Wilczak, *Atlas of Occupational Markers on Human Remains* (Edigrafital, 1999).

123. F. Alves Cardoso, C. Y. Henderson, The categorisation of occupation in identified skeletal collections: A source of bias? *Int. J. Osteoarchaeol.* **23**, 186–196 (2013).

124. K. A. R. Kennedy, Skeletal markers of occupational stress, in *Reconstruction of Life from the Skeleton,* M. Y. İşcan, K. A. R. Kennedy, Eds. (Alan R. Liss Inc., 1989), pp. 129–160.

125. C. Y. Henderson, F. Alves Cardoso, Special issue entheseal changes and occupation: Technical and theoretical advances and their applications. *Int. J. Osteoarchaeol.* **23**, 127–134 (2013).

126. C. Y. Henderson, “Musculo-skeletal stress markers in bioarchaeology: Indicators of activity levels or human variation? A re-analysis and interpretation,” thesis, University of Durham, Durham (2009).

127. S. M. Aguayo, “Variations in skeletal markers and pathologies between Southern Plains equestrian and Puebloan Native American populations,” thesis, Texas Tech University, Lubbock, TX (2012).

128. M. R. Fuka, *Activity markers and horse riding in Mongolia: Entheseal changes among Bronze and Iron Age human skeletal remains (Master thesis)* (Purdue University, West Lafayette, 2018).

129. A. Khudaverdyan, H. Khachatryan, L. Eganyan, The human skeleton from the Late Iron Age burial of Shirakavan (Armenia): A case study. *Bull. Int. Assoc. Paleodontol.* **11**, 51–61 (2017).

130. Š. Anđelinović, I. Anterić, E. Škorić, Ž. Bašić, Skeleton changes induced by horse riding on medieval skeletal remains from Croatia. *Int. J. Hist. Sport* **32**, 708–721 (2015).

131. R. K. Wentz, N. T. de Grummond, Life on horseback: Palaeopathology of two Scythian skeletons from Alexandropol, Ukraine. *Int. J. Osteoarchaeol.* **19**, 107–115 (2009).

132. K. Zejdlik, Z. Nyárádi, A. Gonciar, Evidence of horsemanship in two Szekler noblemen from the Baroque period. *Int. J. Osteoarchaeol.* **31**, 66–76 (2020).

133. C. Baillif-Ducros, G. McGlynn, M. C. Truc, *Cavaliers du passé : Activité et marqueurs ostéologiques. Proposition d'une révision du "Syndrome du cavalier": de L'Europe à l'Asie*. 1839èmes journées de la Société d'Anthropologie de Paris (2014).

134. C. Baillif-Ducros, Christèle, *La pratique de la monte à cheval au haut Moyen Âge (fin Ve - VIIe siècle) dans le nord-est de la Gaule. État des connaissances archéologiques, recherche méthodologique sur le "syndrome du cavalier" et application d'un nouveau protocole d'étude aux populations mérovingiennes* (Université de Caen Normandie, 2018).

135. B. Bühler, S. Kirchengast, A life on horseback? Prevalence and correlation of metric and non-metric traits of the "horse-riding syndrome" in an Avar population (7th-8th century AD) in Eastern Austria. *Anthropol. Rev.* **85**, 67–82 (2022).

136. C. Baillif-Ducros, G. McGlynn, Stirrups and archaeological populations: Bio-anthropological considerations for determining their use based on the skeletons of two Steppe riders. *Bull. Soc. Suisse d'Anthropologie* **19**, 43–44 (2013).

137. G. Tommasini, "Maneggi and Jumps". The basic exercises of Renaissance horsemanship (2014); http://worksofchivalry.com/maneggi-and-jumps-the-basic-exercises-of-renaissance-horsemanship-part-1/ [accessed 19 April 2021].

138. G. Tommasini, "A la brida" and "a la gineta". Different riding techniques in the late Middle Ages and the Renaissance (2014); http://worksofchivalry.com/a-la-brida-and-a-la-gineta-different-riding-techniques-in-the-late-middle-ages-%e2%80%a8and-the-renaissance/ [accessed 19 April 2021].

139. C. Baillif-Ducros, The Merovingian rider and his horse: Impact of equestrian equipment on the rider's posture and skeleton. EAA 2021, Session #509 Horseman-horse couple through time and space (Inrap, 2021).

140. T. A. Tichnell, "Invisible horsewomen: Horse riding and social dynamics on the steppe," thesis, Michigan State University, East Lansing, MI (2012).

141. L. Klejn, The steppe hypothesis of Indo-European origins remains to be proven. *Acta Archaeol.* **88**, 193–204 (2017).

142. V. M. Narasimhan, N. Patterson, P. Moorjani, N. Rohland, R. Bernardos, S. Mallick, I. Lazaridis, N. Nakatsuka, I. Olalde, M. Lipson, A. M. Kim, L. M. Olivieri, A. Coppa, M. Vidale, J. Mallory, V. Moiseyev, E. Kitov, J. Monge, N. Adamski, N. Alex, N. Broomandkhoshbacht, F. Candilio, K. Callan, O. Cheronet, B. J. Culleton, M. Ferry, D. Fernandes, S. Freilich, B. Gamarra, D. Gaudio, M. Hajdinjak, É. Harney, T. K. Harper, D. Keating, A. M. Lawson, M. Mah, K. Mandl, M. Michel, M. Novak, J. Oppenheimer, N. Rai, K. Sirak, V. Slon, K. Stewardson, F. Zalzala, Z. Zhang, G. Akhatov, A. N. Bagashev, A. Bagnera, B. Baitanayev, J. Bendezu-Sarmiento, A. A. Bissembaev, G. L. Bonora, T. T. Chargynov, T. Chikisheva, P. K. Dashkovskiy, A. Derevianko, M. Dobeš, K. Douka, N. Dubova, M. N. Duisengali, D. Enshin, A. Epimakhov, A. V. Fribus, D. Fuller, A. Goryachev, A. Gromov, S. P. Grushin, B. Hanks, M. Judd, E. Kazizov, A. Khokhlov, A. P. Krygin, E. Kupriyanova, P. Kuznetsov, D. Luiselli, F. Maksudov, A. M. Mamedov, T. B. Mamirov, C. Meiklejohn, D. C. Merrett, R. Micheli, O. Mochalov, S. Mustafokulov, A. Nayak, D. Pettener, R. Potts, D. Razhev, M. Rykun, S. Sarno, T. M. Savenkova, K. Sikhymbaeva, S. M. Slepchenko, O. A. Soltobaev, N. Stepanova, S. Svyatko, K. Tabaldiev, M. Teschler-Nicola, A. A. Tishkin, V. V. Tkachev, S. Vasilyev, P. Velemínský, D. Voyakin, A. Yermolayeva, M. Zahir, V. S. Zubkov, A. Zubova, V. S. Shinde, C. Lalueza-Fox, M. Meyer, D. Anthony, N. Boivin, K. Thangaraj, D. J. Kennett, M. Frachetti, R. Pinhasi, D. Reich, The formation of human populations in South and Central Asia. *Science* **365**, eaat7487 (2019).

143. A. V. Poliakov, S. Svyatko, N. F. Stepanova, A review of the radiocarbon dates for the Afanasyevo culture (Central Asia): Shifting towards the “shorter” chronology. *Radiocarbon* **61**, 243–263 (2019).

144. T. R. Hermes, A. A. Tishkin, P. A. Kosintsev, N. F. Stepanova, B. Krause-Kyora, C. A. Makarewicz, Mitochondrial DNA of domesticated sheep confirms pastoralist component of Afanasievo subsistence economy in the Altai Mountains (3300-2900 cal BC). *Archaeol. Res Asia* **24**, 100232 (2020).

145. E. B. Vadetskaia, A. V. Poliakov, N. F. Stepanova, *Svod pamiatnikov afanas'evskoi kul'tury* (Azbuka, 2014).

146. V. Heyd, Yamnaya groups and tumuli west of the Black Sea, in *Ancestral Landscapes in Ancestral Landscape. Burial mounds in the Copper and Bronze Ages. Proceedings of the International Conference held in Udine, May 15th–18th 2008*, E. Borgna, S. Müller Celka, Eds. (Travaux de la Maison de l'Orient et de la Méditerranée, Série recherches archéologiques 58, 2011), pp. 535–555.

147. I. Mathieson, S. Alpaslan-Roodenberg, C. Posth, A. Szécsényi-Nagy, N. Rohland, S. Mallick, I. Olalde, N. Broomandkhoshbacht, F. Candilio, O. Cheronet, D. Fernandes, M. Ferry, B. Gamarra, G. González Fortes, W. Haak, E. Harney, E. Jones, D. Keating, B. Krause-Kyora, I. Kucukkalipci, M. Michel, A. Mittnik, K. Nägele, M. Novak, J. Oppenheimer, N. Patterson, S. Pfrengle, K. Sirak, K. Stewardson, S. Vai, S. Alexandrov, K. W. Alt, R. Andreescu, D. Antonović, A. Ash, N. Atanassova, K. Bacvarov, M. Balázs Gusztáv, H. Bocherens, M. Bolus, A. Boroneant, Y. Boyadzhiev, A. Budnik, J. Burmaz, S. Chohadzhiev, N. J. Conard, R. Cottiaux, M. Čuka, C. Cupillard, D. G. Drucker, N. Elanski, M. Francken, B. Galabova, G. Gantesovski, B. Gély, T. Hajdu, V. Handzhyiska, K. Harvati, T. Higham, S. Iliev, I. Jankovič, I. Karavanič, D. J. Kennet, D. Kamso, A. Kozak, D. Labuda, M. Lari, C. Lazăr, M. Leppek, K. Leshtakov, D. Lo Vetro, D. Los, I. Lozanov, M. Malina, F. Martini, K. Mcsweeney, H. Meller, M. Mandušič, P. Mirea, V. Moiseyev, V. Petrova, T. D. Price, A. Simalcsik, L. Sineo, M. Šlaus, V. Slavchev, P. Stanev, A. Starovič, T. Szeniczey, S. Talamo, M. Teschler-Nicola, C. Thevenet, I. Valchev, F. Valentin, S. Vasilyev, F.

Veljanovska, S. Venelinova, E. Veselovskaua, B. Viola, C. Virag, J. Zaninovič, S. Zäuner, P. W. Stockhammer, G. Catalano, R. Krauß, D. Caramelli, G. Zarina, B. Gaydarska, M. Lillie, A. G. Nikitin, I. Potekhina, A. Papathanasiou, D. Borič, C. Bonsall, J. Krause, R. Pinhasi, D. Reich, The genomic history of southeastern Europe. *Nature* **555**, 197–203 (2018).

148. J. Dani, G. Kulcsár, Yamnaya interactions in the Carpathian Basin, in *Yamnaya Interactions. Proceedings of the International Workshop held in Helsinki, 25–26 April 2019*, V. Heyd, G. Kulcsár, B. Preda-Bălănică, Eds. (The Yamnaya Impact of Prehistoric Europe 2, Archaeolingua, 2021), pp. 329–359.

149. E. Kaiser, K. Winger, Pit graves in Bulgaria and the Yamnaya Culture. *Präehistorische Zeitschrift* **90**, 1–27 (2015).

150. S. Alexandrov, Bronze Age barrow graves in Upper Thrace—Old and new questions, in *Repräsentationen der Macht, Beiträge des Festkolloquiums zu Ehren des 65. Geburtstags von Blagoje Govedarica*, S. Hansen, Ed. (Harrasowitz Verlag Wiesbaden, 2020), pp. 147–170.

151. A. Frînculeasa, The Children of the Steppe: Descendance as a key to Yamnaya success. *Studii de Preistorie* **16**, 129–168 (2019).

152. J. Koledin, U. Bugaj, P. Jarosz, M. Novak, M. M. Przybyła, M. Podsiadło, A. Szczepanek, M. Spasić, P. Włodarczak, First archaeological investigations of barrows in the Bačka region and the question of the Eneolithic/Early Bronze Age barrows in Vojvodina. *Präehistorische Zeitschrift* **95**, 350–375 (2020).

153. A. Frînculeasa, M. Frînculeasa, I. Dumitru, C. Buterez, The dynamics of prehistoric burial mounds of Ploieşti metropolitan area (Romania) as reflected by cartographic documents of the 18th-20th centuries. *Area* **49**, 533–544 (2017).

154. A. Frînculeasa, Burial mounds in the Lower Danube region. From the international to the local and the other way round, in *Yamnaya Interactions. Proceedings of the International*

*Workshop held in Helsinki, 25–26 April 2019*, V. Heyd, G. Kulcsár, B. Preda-Bălănică, Eds. (The Yamnaya Impact of Prehistoric Europe 2, Archaeolingua, 2021), pp. 173–205.

155. A. Frînculeasa, Earthen burial mounds and the Coțofeni culture south of the Carpathians. Archaeological research in Ariceștii-Rahtivani-Movila pe Răzoare. *Ziridava. Studia Archaeol.* **34**, 35–90 (2020).

156. A. Frînculeasa, B. Preda, O. Negrea, A. Soficaru, V. Dumitraşcu, M. Frînculeasa, Complexe funerare de la începutul mileniului al II-lea descoperite recent în judeţul Prahova. *Mater. și Cercet. Arheol.* **8**, 139–163 (2012).

157. C. Bronk Ramsey, S. Lee, Recent and planned developments of the program OxCal. *Radiocarbon* **55**, 720–730 (2013).

158. A. Frînculeasa, B. Preda, C. Dumitrescu, O. Negrea, A.-D. Soficaru, Blejoi, jud. Prahova, in *Cronica Cercetărilor Arheologice din România, Campania 2016, A LI-a Sesiune Națională de Rapoarte Arheologice, Muzeul Național de Istorie a României, București 24–27 Mai 2017* (Institutul Naţional al Patrimoniului, 2017), pp. 166–168.

159. C. Schuster, A. Morintz, R. Băjenaru, A. Ioniţă, A. Măgureanu, C. Ştefan, A. Popescu, D. Sârbu, D. Măgureanu, R Kogălniceanu, E. Dumitraşcu, M. Vasile, M. Constantin, C. Constantin, Peştera, com. Peştera, jud. Constanţa, "Peştera, com. Peştera, jud. Constanţa, Punct: Km 168+500–167+700 (Tumulul nr. 5 şi 6), km 168+600–169+100 (Tumulul nr. 3) şi km 169+800–171+000 (Valul mic de pământ)" in *Cronica Cercetărilor Arheologice din România. Campania 2010, A XLIII-a Sesiune Naţională de Rapoarte Arheologice, Sibiu, 26–29 mai 2011* (Muzeul Brukenthal, 2011), pp. 215–217.

160. P. Włodarczak, T. Valchev, От степите към Балканите ...Проучване на надгробни могили в област Ямбол/Ze tepów na Bałkany...Badania kurhanów w obwodzie Jamboł/From the steppes to the Balkans...Excavation on burial mounds in Yambol district (Regional Museum of History Yambol Institute of Archaeology and Ethnology of the Polish Academy of Sciences, 2022).

161. S. Alexandrov, V. Slavchev, E. Tonkova, Rescue excavations of Bronze Age barrows in Vetrino region, northeast Bulgaria. *Mater. şi Cercet. Arheol.* **17**, 5–48 (2021).

162. Gy. Gazdapusztai, Zur Frage der Verbreitung der sogenannten “Ockergräberkultur” in Ungarn. *Móra Ferenc Múz. Évk.* **65**, 31–38 (1963).

163. I. Ecsedy, *The People of the Pit-Grave Kurgan in Eastern Hungary* (Fontes Archaeologici Hungariae, Akad. Kiadó, 1979).

164. T. Horváth, J. Dani, Á. Petö, L. Pospiezny, É. Svingor, Multidisciplinary contributions to the study of pit grave culture Kurgans of the Great Hungarian Plain, in *Transitions to the Bronze Bronze Age. Interregional Interaction and Socio-Cultural Change in the Third Millennium BC Carpathian Basin and Neighbouring Regions*, V. Heyd, G. Kulcsár, V. Szeverényi, Eds. (Archaeolingua, 2013), pp. 153–179.

165. C. Gerling, *Prehistoric Mobility and Diet in the West Eurasian Steppes 3500 to 300 BC. An isotopic Approach* (Topoi Berlin Studies of the Ancient World 25, De Gruyter, 2015).

166. K. Nagy, Csongrád-Kettőshalom-Petőfi Tsz. *Régészeti Füzetek* Ser. **I**/17 (Budapest, 1964), pp. 14.

167. I. Ecsedy, A new item relating the connections with the East in the Hungarian Copper Age (a Marosdécse type grave in Csongrád). *A Móra Ferenc Múzeum Évkönyve* **1971-2**, 9–17 (1974).

168. J. Dani, T. Horváth, *Őskori kurgánok a magyar Alföldön. A Gödörsíros (Jamnaja) entitás magyarországi kutatása az elmúlt 30 év során. Áttekintés és revízió* (Archaeolingua Kiadó, 2012).

169. A. Marcsik, Data of the Copper Age anthropological find of Bárdos-farmstead at Csongrád-Kettőshalom. *A Móra Ferenc Múzeum Évkönyve* **1971-2**, 19–27 (1974).

170. I. Ecsedy, Eine neue Hügelbestattung der “Grubengrab-Kultur” (Kupferzeit-Frühbronzezeit) in Dévaványa (Vorbericht). Archäologische Forschungen 1969. *Antaeus – Mitteilungen des Archäologischen Instituts der Ungarischen Akademie der Wissenschaften* **2**, 45–50 (1971).

171. MRT 6: I. Ecsedy, L. Kovács, B. Maráz, I. Torma, *Magyarország Régészeti Topográfiája 6. Békés megye régészeti topográfiája. A szeghalmi járás IV/1* (Akadémiai Kiadó, 1982).

172. Á. Bede, *A tiszántúli halmok régészeti geológiai és környezettörténeti szempontú vizsgálati lehetőségei* (Doctoral Dissertation, Department of Geology and Palaeontology, University of Szeged, Hungary, 2014); http://doktori.bibl.u-szeged.hu/id/eprint/2447/17/Bede%20Adam-tezisfuzet_magyar.pdf [accessed 20 March 2022].

173. Á. Bede, Beszámoló a Békés megyei Nagy-Sárrét halmainak felméréséről. (Report on mound survey in the Nagy-Sárrét region (Békés county, Hungary). *Crisicum* **8**, 17–43 (2014).

174. A. Marcsik, The anthropological material of the pit-grave kurgans in Hungary, in *The People of the Pit-Grave Kurgans in Eastern Hungary*, I. Ecsedy, Ed. (Fontes Archaeologici Hungariae, Akadémiai Kiadó, 1979), pp. 87–98.
